# Tunable aryl alkyl pyrazolium tetrafluoroborate ionic liquids/salts: synthesis, characterization, and applications for removal of methyl orange from aqueous solution

**DOI:** 10.3906/kim-2106-67

**Published:** 2021-09-27

**Authors:** Melek CANBULAT ÖZDEMİR

**Affiliations:** Department of Environmental Engineering, Faculty of Engineering, Middle East Technical University, Ankara, Turkey

**Keywords:** Tunable aryl alkyl ionic liquids, pyrazolium salts, tetrafluoroborate anion, methyl orange removal

## Abstract

In this work, new tunable aryl alkyl pyrazolium tetrafluoroborate ionic liquids/salts, 2-ethyl-1-(p-X-phenyl)-3,5-dimethylpyrazolium tetrafluoroborate [X: −Br (4a), −OCH_3_ (4b), −NO_2_ (4c)] and 2-butyl-1-(p-X-phenyl)-3,5-dimethylpyrazolium tetrafluoroborate [X: −Br (5a), −OCH_3_ (5b), −NO_2_ (5c)], were synthesized by following halide-free synthetic route. Their chemical structures were identified through NMR (^1^H, ^13^C, ^19^F), IR, elemental analysis, and HRMS data. The synthesized 4a–4c and 5a–5c salts were used for the removal studies of methyl orange dye from aqueous solutions. The effects of specific parameters such as nature of the solvent, pH, contact time, amount and structure of the salts, and concentration of potassium chloride on the removal efficiencies were investigated. Experimental results revealed that methyl orange could be removed from the aqueous solution up to 99.7% under the optimized conditions. The composition of the ion pairs between the cation of the 4b and anion of methyl orange was determined. The reuse of the 4b was achieved up to five cycles, with high extraction efficiencies of over 90 %. Accordingly, a time-efficient, simple, and highly effective method has been presented to remove methyl orange dye from aqueous solutions.

## 1. Introduction

The organic salts, comprised solely of ions and having melting temperatures lower than 100 °C, are recognized as ionic liquids (ILs) [1]. The existing ILs consist predominantly of 1,3-dialkylsubstituted imidazolium cations and organic/inorganic anions (CH_3_COO^−^, CH_3_SO_3_^−^, HSO_4_^−^, Cl^−^, BF_4_^−^, PF_6_^−^). Although pyrazole is the structural isomer of the imidazole structure, the number of studies associated with pyrazolium based ILs is quite limited compared to imidazolium-based ILs [2–7]. The synthesis of ILs is generally carried out by following a two-step synthetic procedure. The quaternization reaction is the first step for synthesizing ILs with desired cations using appropriate alkylating agents such as alkyl halides, alkyl methanesulfonates, dialkyl sulfates, and dimethyl carbonate. Lately, the synthesis of halide-free ILs has attracted attention because of the difficulties encountered in removing halide impurities, which affect the properties of ILs. The optional second step of the IL synthesis is anion metathesis or anion exchange reactions to obtain ionic liquids with desired anions [8–14].

Ionic liquids with intriguing properties have found an extensive range of applications in diverse fields [15–20]. The characteristics of ILs could easily be adjusted by changing the combination of cations and anions. Accordingly, a new type of ILs containing an aryl ring on their cations, specified as tunable aryl alkyl ionic liquids (TAAILs), has been developed recently. The properties of TAAILs could be changed by electronic and steric effects of the substituent at the aryl ring [10, 21–25].

Synthetic dyes generally used in textile, cosmetic, plastic, food, and drug industries cause a generation of large amounts of dye-polluted water bodies worldwide. Thus, many studies, including chemical, physical, and biological methods, have been conducted for removing dyes from water bodies [26–30]. Methyl orange (MO), generally used as an acid-base indicator, is an anionic, water-soluble azo group of synthetic dye. The sequestration of MO from water bodies is an issue of interest for environmental sciences due to the presence of the azo group that causes the mutagenic and carcinogenic degradation products under anaerobic conditions and its low biodegradability [20, 31–35].

In this work, newly synthesized tunable aryl alkyl pyrazolium tetrafluoroborate ionic liquids/salts (4a–4c, 5a–5c) were applied as an extractant for removing MO from the aqueous solution. The effects of the nature of the solvent, pH, contact time, amount and structure of the TAAILs, and concentration of potassium chloride (KCl) on the extraction efficiencies were investigated. In addition, the composition of ion pairs between the 4b and MO was determined spectrophotometrically using Job’s method of continuous variations. Furthermore, from an economic perspective, the reusability of the 4b salt was examined.

## 2. Materials and methods

### 2.1. Materials

All chemical materials were supplied commercially and used as received. Arylhydrazinium hydrochloride derivatives, acetylacetone, ethyl methanesulfonate, ethyl acetate, acetic acid (glacial), ethanol, n-hexane, acetonitrile, methyl orange (4-dimethylaminoazobenzene-4’-sulfonic acid sodium salt), sodium chloride were obtained from Merck. Dichloromethane, n-butanol, diethyl ether, methanesulfonyl chloride, sodium bicarbonate, sodium sulfate, tetrafluoroboric acid (HBF_4_, 48 wt.% in H_2_O) were acquired from Sigma-Aldrich. Butyl methanesulfonate, and 3,5-dimethyl-1-(p-X-phenyl)-1H-pyrazoles [X: −Br (1a), −OCH_3_ (1b), −NO_2_ (1c)] were synthesized according to the previous reports [36, 10].

Ultrapure water obtained from Millipore Rios 16 water purification system was used for through-out the experiments. The microwave-assisted synthesis of 1a–1c, 2a–2c, and 3a–3c compounds was performed using a “Microsynth-Milestone” multimode oven. The IR spectra were obtained by a “Thermo Fischer Scientific Nicolet iS10” spectrometer. NMR spectra (^1^H, ^13^C, and ^19^F) of the TAAILs were acquired by a “Bruker Ultrashield 300 MHz” NMR spectrometer. The elemental analyses were conducted with a “LECO CHNS-932” elemental analyzer. High-resolution mass spectrometry (HRMS) data were acquired on a Bruker Daltonics maXis II ETD nLC/LC-QTOF mass spectrometer using electrospray ionization (ESI) technique in positive mode. The melting points of the salts were detected with an “Electrothermal 9200” melting point apparatus. The maximum absorption wavelength of the ion pairs between the synthesized salts and MO was ascertained with a “Perkin Elmer Lambda 25” UV-Vis spectrophotometer. The pH and absorbance values of the methyl orange solutions were measured with an “Oakton pH 450” digital pH meter and “Hach DR 3900” UV-Vis spectrophotometer (464 nm), respectively.

### 2.2. General synthetic procedure for 2a–2c and 3a–3c

The 2-ethyl-1-(p-X-phenyl)-3,5-dimethylpyrazolium methanesulfonate [X: −Br (2a), −OCH_3_ (2b), −NO_2_ (2c)] and 2-butyl-1-(p-X-phenyl)-3,5-dimethylpyrazolium methanesulfonate [X: −Br (3a), −OCH_3_ (3b), −NO_2_ (3c)] salts were synthesized by following the reported procedure [30]. The 2c and 3c compounds have been synthesized for the first time in this study. In brief, the relevant pyrazole compound (1a–1c) was dissolved in acetonitrile. An alkyl methanesulfonate derivative (ethyl methanesulfonate (CH_3_SO_3_C_2_H_5_) for 2a–2c; butyl methanesulfonate (CH_3_SO_3_C_4_H_9_) for 3a–3c), equivalent mole, was added to this solution. Then, the reaction vessel was irradiated by MW at 80 °C for 30 min. The TAAILs with methanesulfonate anion obtained were purified as described and dried under vacuum. The obtained spectral data of 2a–2c and 3a–3c salts were presented in Supplementary Materials.

### 2.3. General synthetic procedure for 4a–4c and 5a–5c

The synthesis of 2-ethyl-1-(p-X-phenyl)-3,5-dimethylpyrazolium tetrafluoroborate [X: −Br (4a), −OCH_3_ (4b), −NO_2_ (4c)] and 2-butyl-1-(p-X-phenyl)-3,5-dimethylpyrazolium tetrafluoroborate [X: −Br (5a), −OCH_3_ (5b), −NO_2_ (5c)] TAAILs were carried out following the described procedure [10]. In brief, the appropriate salt (2a–2c, 3a–3c) was dissolved in ultrapure water. The equivalent mole of HBF_4_(aq) (48% in water) was slowly added under constant stirring to this solution, and then it was continued to stir for 2 h. The solid formed was filtered off and crystallized in ethanol. The obtained spectral data of the 4a–4c, 5a–5c salts were presented in Supplementary Materials.

### 2.4. The removal procedure of methyl orange

A known amount of the salt (4a–4c, 5a–5c) was added to the methyl orange solution (3 mL) in the rubber stoppered vial and stirred at 150 rpm at room temperature during the specified time. The obtained solution was transferred into a separating funnel, and dichloromethane (3 mL) was added. The funnel was gently swirled until the liquids were adequately mixed by releasing excess pressure several times. Then, the funnel was placed in the ring stand and waited until two separate layers were formed. Finally, the absorbance of the separated aqueous phase was measured.

The removal efficiencies (%) were calculated with equations (Eq (1) and Eq (2)) which are given below:


(1) $$D=\frac{C_{i}-C_{f}}{C_{f}}\times \frac{V_{aq}}{V_{org}}$$

(2) $$Removal\;efficiency\;(\%)=\frac{D}{D+\frac{V_{aq}}{V_{org}}}\times 100$$

In these equations, D indicates the distribution ratio of MO between the organic and the aqueous phase; C_i_ and C_f_ indicate the initial and final concentrations of MO; Vaq and Vorg indicate the volumes of the aqueous and the organic phase. The measured volumes of aqueous and organic phases before and after extraction experiments were approximately the same due to the low miscibility of dichloromethane in water. The removal experiments of MO were performed two times, and an average of the obtained values was presented. The calibration curve was obtained with a correlation coefficient of 0.9999 (Supplementary Materials Figure S1).

## 3. Results

### 3.1. Synthesis and characterization

**Figure 1:**
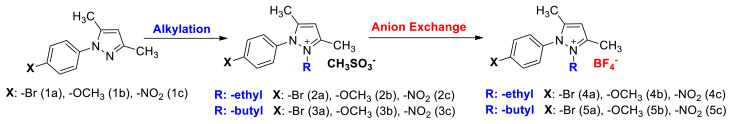
Synthetic route for tunable aryl alkyl pyrazolium tetrafluoroborate ionic liquids/salts (Alkylation: CH_3_SO_3_C_2_H_5_ (for 2a–2c); CH_3_SO_3_C_4_H_9_ (for 3a–3c)_,_ MW, 80 °C, in CH_3_CN; Anion exchange: HBF_4_(aq), R.T, in H_2_O).

The usage of alkyl halides causes several problems such as low yields, harsh reaction conditions, and difficulties in removing halide impurities, which affect the properties of ILs [9]. As shown in Figure 1, 2a–2c, and 3a–3c salts were synthesized by the alkylation of 1a–1c compounds with ethyl and butyl methanesulfonate under MW irradiation. The applied halide-free synthetic procedure for synthesizing methanesulfonate salts eliminates potential problems associated with alkyl halide usage for quaternization reactions. Additionally, the application of MW irradiation provides advantages such as short reaction times, high yield, and minimum by-products [37]. The TAAILs (4a–4c and 5a–5c) were synthesized using their corresponding methanesulfonate congeners (2a–2c, 3a–3c) and HBF_4_(aq) (Figure 1).

The obtained spectral data, elemental analysis, and HRMS results were congruent with the chemical structures of the salts (Supplementary Materials). The spectral differences between ethyl substituted 4a–4c salts and butyl substituted 5a–5c salts were observed, compatible with their chemical structures.

The aromatic ν(C-H) bands, and ν(C=C) and ν(C=N) bands of 2-alkyl-1-(p-X-phenyl)-3,5-dimethylpyrazolium cations were observed at 3070–3103 cm^−1^ and 1650–1411 cm^−^^1^ range in their IR spectra. The asymmetric and symmetric ν(C-H) stretching vibrations of alkyl groups were seen between 2995 and 2848 cm^−^^1^. In the IR spectra of p-Br substituted 4a and 5a salts, the bands assigned to ν(C-Br) vibrations were observed at 652 cm^−^^1^ and 679 cm^−^^1^. The ν(C-O) stretching vibrations of p-OCH_3_ substituted 4b, and 5b salts appeared at 1251 cm^−^^1^ and 1261 cm^−^^1^. The asymmetric ν(N-O) stretching vibrations for the p-NO_2_ substituted 4c, and 5c salts were observed at 1527 cm^−^^1^ and 1537 cm^−^^1^ as a strong band. In addition, the symmetric ν(N-O) stretching vibrations of the 4c and 5c were seen at 1357 cm^−^^1^ and 1355 cm^−^^1^. The very strong bands observed between 1033 cm^−^^1^ and 1037 cm^−^^1^ were attributed to the BF_4_^−^ anion of the TAAILs [38].

**Figure 2:**
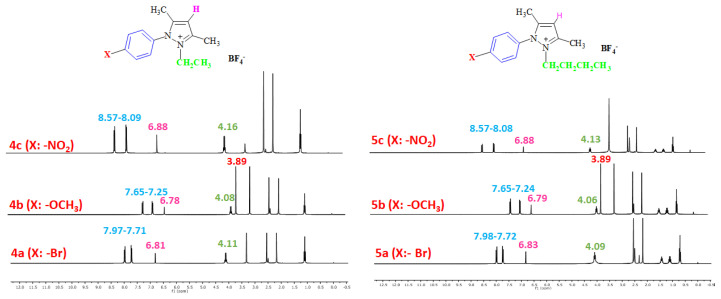
The ^1^H NMR spectra of the 4a–4c and 5a–5c salts.

The aromatic protons were observed as doublets in the range of δ 7.24–8.57 ppm, depending on the nature of the p-substituent, in the ^1^H NMR spectra of the TAAILs (Figure 2). Accordingly, the protons of the aryl ring for p-OCH_3_ substituted salts shift to the upfield regions of ^1^H NMR spectra while the protons of the aryl ring for p-NO_2_ and p-Br substituted salts shift to the downfield regions of ^1^H NMR spectra. As seen in Figure 2, the same trend was observed for the −CH proton of the pyrazolium cation, and the peaks shifted depending on their p-substituent (δ 6.78–6.88). The quartet peaks seen at *δ* 4.08–4.16 ppm were attributed to the −NCH_2_ protons of ethyl group for 4a–4c salts, and the triplet peaks observed at *δ* 4.06–4.13 ppm were attributed to the −NCH_2_ protons of butyl group for 5a–5c salts. The −CH_3_ protons of ethyl alkyl chain for 4a–4c salts were seen at *δ* 1.10–1.11 ppm as a triplet. The −CH_3_ protons of the butyl alkyl chain for 5a–5c salts were seen at δ 0.71 ppm as a triplet. The Ph-OCH_3_ protons for 4b and 5b salts were seen at *δ* 3.89 ppm as a singlet. The −CH_3_ protons at 5 and 3 positions of pyrazolium cations of the TAAILs appeared in the range of *δ* 2.16–2.22 ppm and *δ* 2.54–2.59 ppm as a singlet.

The obtained data from the ^1^H NMR spectra were supported by the ^13^C NMR spectra of the salts. The ^13^C NMR spectra of the TAAILs exhibited 11 signals for 4a and 4c salts, 12 signals for 4b salt, 13 signals for 5a and 5c salts, and 14 signals for 5b salt. The carbon atom of the methoxy group for 4b and 5b salts were seen at *δ* 56.25 ppm and 55.68 ppm. The peaks observed at *δ* 107.64–109.07 ppm correspond to the −CH carbon atom of pyrazolium cation. The peaks of aromatic carbon atoms of the synthesized salts appeared between *δ* 126.18 and 150.05 ppm, except for 4b and 5b salts. The peaks at *δ* 162.19 ppm and *δ* 161.58 ppm were attributed to the p-OCH_3_ substituted aromatic carbon atom of aryl ring for 4b and 5b salts. Two singlet peaks at ca. *δ* (–148.31) and (–148.21) ppm were observed in the ^19^F NMR spectra of 4a–4c and 5a–5c salts, due to the isotopes of the boron atom.

### 3.2. Melting points

**Figure 3:**
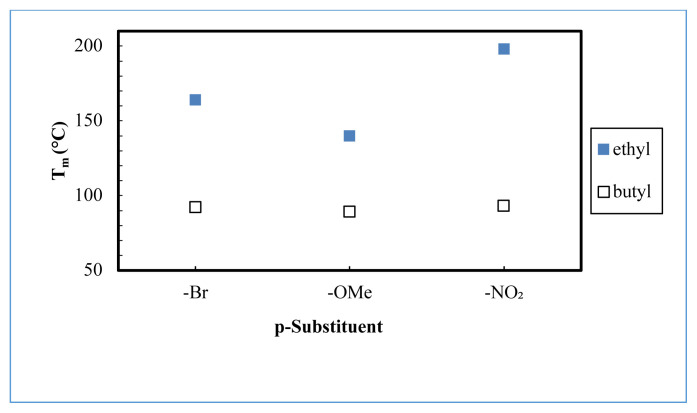
Melting points of tunable aryl alkyl pyrazolium tetrafluoroborates (4a–4c, 5a–5c).

Based on the obtained results, 5a–5c salts have melting temperatures below 100 °C and can be considered ionic liquids. However, the 4a–4c salts have relatively high melting temperatures above 100 °C and do not meet the ionic liquid criteria (Figure 3). Furthermore, it was observed that the electron-donating/withdrawing character of the p-substituent and the length of the alkyl chain affect their melting points. However, the effect of the alkyl chain is remarkable than that of the p-substituent (Figure 3). Accordingly, the melting points of butyl substituted salts (5a–5c) are lower than the melting points of ethyl substituted salts (4a–4c) for the same p-substituent. Besides, the melting temperatures of the salts are ordered from low to high as follows: p-OMe, p-Br, and p-NO_2_ for the same alkyl chain length.

### 3.3. Removal of methyl orange

The effects of the nature of the solvent, pH, contact time, structure and amount of the TAAILs, and KCl concentration on the removal efficiencies of MO, using synthesized TAAILs as an extractant, have been studied.

#### 3.3.1. Effect of nature of solvent, pH, and contact time

The influence of the solvent type on the removal efficiencies was examined according to the described extraction procedure by using different solvents. The 4b salt was used as an extractant with a 0.02 MO/TAAIL molar ratio to remove the MO (initial concentration of 0.54 mmol/L) from an aqueous solution. The obtained removal efficiencies with dichloromethane, chloroform, ethyl acetate, and hexane were 99.2%, 97.9%, 36.4%, and 5.7%, respectively. Due to its highest extraction efficiency, dichloromethane was chosen as a suitable solvent for subsequent extraction processes.

**Figure 4:**
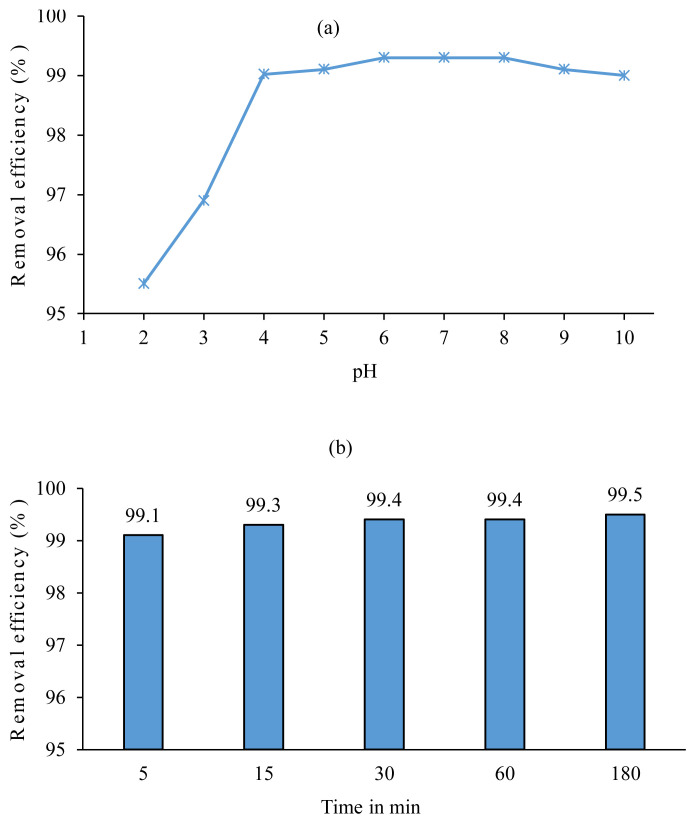
(a) The effect of pH on the removal efficiencies (MO concentration: 0.54 mmol/L; MO/TAAIL (4b) ratio: 0.02) (b) Effect of contact time (MO concentration: 0.54 mmol/L, MO/TAAIL (4a) ratio: 0.02, Volume of aqueous phase: 3 mL, Volume of dichloromethane: 3 mL).

It is well known that the ionization degree of methyl orange depends on the pH of the aqueous phase. Accordingly, MO can be found in two distinct forms: (1) the deprotonated form (pH > pKa (3.46), anionic form, yellow color), (2) protonated form (pH < pKa, zwitterionic form, red color) (Supplementary Materials Figure S2) [39–41]. Therefore, the extraction experiments were carried out in the pH range of 2–10. As presented in Figure 4a, the maximum removal efficiencies of MO were obtained in the pH range of 6–8. At pH 2 and 3, the removal efficiencies of MO decreased by about 3.5% and 2.1%, respectively, compared to pH ≥ 4. On the other hand, above pH 8, the removal efficiencies slightly decreased. However, in pH range 4–10, the removal efficiencies of MO are higher than 99.0%, and the results are close to each other.

The effect of contact time was investigated by conducting experiments ranging the contact time from 5 to 180 min by using 4a salt (as an extractant) and dichloromethane (solvent). As seen in Figure 4b, after 5 min, 99.1%, and after 30 min, 99.4% of MO is removed from the aqueous solution. Nevertheless, the removal efficiency increased by only 0.1% (99.5%) following the increasing contact time to 180 min. Hence, the subsequent experiments were conducted for a contact time of 30 min.

#### 3.3.2. Effect of amount and structure of the TAAILs

Two series of experiments were performed by varying MO/TAAIL molar ratios to investigate the effect of the amount of TAAILs on the removal efficiencies. All experiments were conducted using the MO solution with an initial concentration of 0.54 mmol/L, and in the absence of TAAILs, only 3% of MO was extracted to the dichloromethane phase. The pH of the solutions was adjusted between 4~4.5 for only 5a and 5c salts. All other experiments were conducted at the natural pH values of the salts (pH:4~4.5).

**Figure 5:**
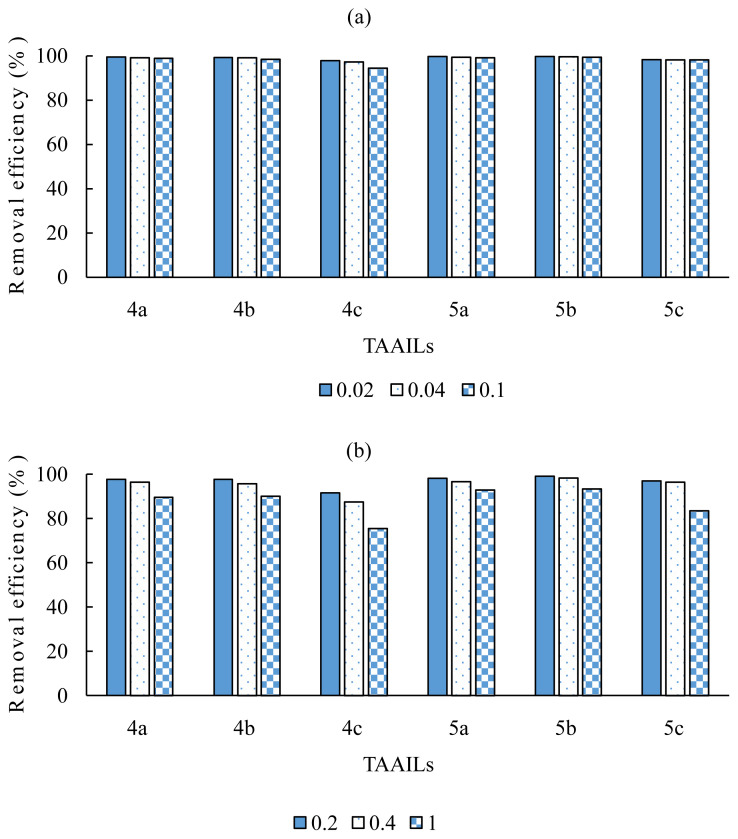
The removal efficiencies of the TAAILs; (a) with 0.02, 0.04, and 0.1 MO/TAAIL molar ratios (b) with 0.2, 0.4, and 1.0 MO/TAAIL molar ratios.

In the first series, the experiments were performed with 0.02, 0.04, and 0.1 MO/TAAIL molar ratios (Figure 5a, Supplementary Materials Table S1). The analysis of the separated water phase reveals that the removal of MO is achieved with high efficiencies up to 99.7%. It is also remarkable that the butyl substituted TAAILs show slightly higher removal efficiencies than their ethyl substituted counterparts. Also, the removal efficiencies obtained using p-Br and p-OCH_3_ substituted TAAILs are close to each other and somewhat higher than p-NO_2_ substituted TAAILs.

In the second series of experiments, relatively high MO/TAAIL molar ratios (0.2, 0.4, 1.0) were implemented (Figure 5b, Supplementary Materials Table S2). As seen in Figure 5b, the removal efficiencies of MO stay high up to a MO/TAAIL ratio of 0.4, though decrease at about 10% for an equimolar MO/TAAIL amounts. Additionally, the same trend described for low MO/TAAILs ratios based on the structure of the TAAILs was also observed for relatively high MO/TAAIL molar ratios.

#### 3.3.3. Effect of KCl concentration

Generally, salts and dyes exist together in the actual textile dye effluents. Thus, the effect of salt concentration on the removal efficiencies was investigated by dissolving the appropriate amount of KCl in the aqueous medium to obtain varying concentrations between 3 and 30% w/v. As a result, the extraction efficiency of MO increased to 99.9% by increasing the KCl concentration to 24% w/v and stayed constant for 30% w/v KCl concentration (Supplementary Materials Figure S3). Increased removal efficiencies by adding KCl to the aqueous phase can be explained by the reduced degree of hydration of MO in solutions, as declared in previous studies [35,42].

### 3.4. Reuse of the TAAILs

**Figure 6:**
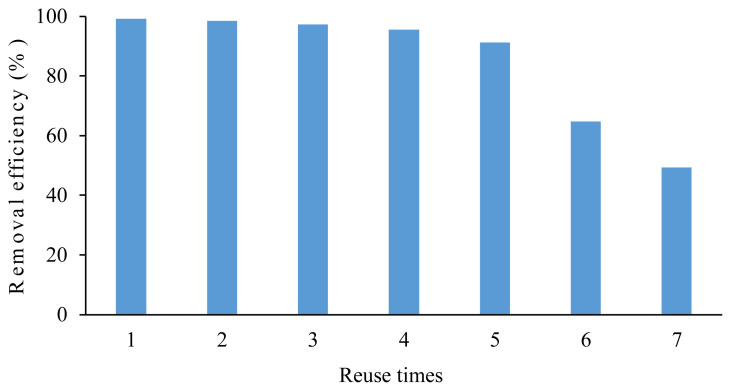
The reuse of 4b salt to remove MO from aqueous solution.

The reuse of ILs is a substantial issue for green and sustainable chemistry. The results mentioned above indicate that the removal efficiencies are higher than 99.0% in the range of pH 4–10. Therefore, separating MO from TAAILs by changing the pH values of solutions is difficult [43]. Hence, the synthesized TAAIL was used directly for the next cycle of extraction. The 4b salt was examined as a model for the extraction of MO (0.54 mmol/L) from an aqueous solution with a 0.02 MO/IL molar ratio. The first cycle of the experiment was conducted by following the same extraction procedure described above. Then, the organic phase (4b salt+CH_2_Cl_2_) was reused for the next cycle of the experiment. As shown in Figure 6, the removal efficiency of 4b salt remained high up to 91% after the fifth use. However, the removal efficiencies of MO decreased in sixth and seventh use to 65% and 49%.

### 3.5. Formation and stoichiometry of ion pairs

The formation of ion pairs between the TAAILs and MO was studied spectrophotometrically. A hypsochromic shift (from 424 nm to 418 nm) and a hyperchromic shift were seen for the UV band of MO in dichloromethane (Supplementary Materials Figure S4). Furthermore, similar changes were observed for the UV spectra of the other salts (Supplementary Materials Figure S5). These widely recognized spectral changes that appeared for the UV band of MO indicate the formation of ion pairs between the TAAILs and MO [44, 45].

The composition of the ion pairs between the TAAIL (4b) and MO was determined by employing Job’s method of continuous variations [46]. First, a series of solutions were prepared by keeping the total volume at 10 mL using equimolar aqueous solutions of the 4b salt and MO (0.27 mmol/L). The solutions were stirred at 150 rpm for 30 min, then dichloromethane (10 mL) was added. The absorbance of the organic phase was measured at the maximum wavelength (418 nm) of the ion pairs formed between the 4b salt and MO. The maximum absorbance value was observed at a mole fraction of 0.5, indicating the ion pairs formation between the 4b and MO in a stoichiometric ratio of 1:1 (Supplementary Materials Figure S6).

## 4. Conclusion

In conclusion, six new tunable aryl alkyl pyrazolium tetrafluoroborate ionic liquids/salts were synthesized by following a halide-free synthetic procedure. Their structures were determined by appropriate spectroscopic methods. The removal of MO from an aqueous solution with newly synthesized TAAILs was studied in detail. The obtained results reveal that the synthesized salts could effectively be used as an extractant for the removal of MO from an aqueous solution. The acquired removal efficiencies are relatively high in the pH range of 4–10 and are affected by the amount and structure of the TAAILs and the concentration of KCl. The reusability studies show that the 4b salt could be reused five times with high removal efficiencies of over 90% without any additional process. The spectrophotometric studies reveal that the ion pairs between MO and TAAILs are formed in a stoichiometric ratio of 1:1. It is well known that removing synthetic dyes from wastewaters is of great importance for a sustainable environment. In this context, the present method, which has the advantages of simplicity, the high removal efficiency of MO, and time efficiency, might be a promising alternative for removing anionic dyes from the wastewaters.

## Supplementary Material

**Table:** 

**Table of Contents**	
**(28 Pages)**	
The spectroscopic analysis data of 2a–2c, 3a–3c, and ^1^H NMR and ^13^C NMR spectra of 2c and 3c compounds……………..………...………………………………….................................…	S1–S5
IR, ^1^H-NMR, ^13^C NMR, ^19^F NMR, and HRMS spectra of 4a–4c, 5a–5c................................	S6–S23
Calibration curve of methyl orange (Figure S1)..........................................................................	S24
The main structures of MO in solutions (A) anionic form (B) zwitterionic form (Figure S2)…..	S24
The removal efficiencies of TAAILs with 0.02, 0.04, and 0.1 MO/TAAIL molar ratios (Table 1)....................................................................................................................................	S25
The removal efficiencies of TAAILs with 0.2, 0.4, and 1.0 MO/TAAIL molar ratios (Table S2)…………………………………………………………………………….………..	S26
Effect of KCI concentration on the removal efficiencies (Figure S3)…………………………..	S27
Molecular absorption spectra of (1) MO and (2) 4b: MO ion pairs in dichloromethane (Figure S4)…………………....………………………………………………………………..	S27
Molecular absorption spectra of TAAIL: MO ion pairs (Figure S5)………………..…….……	S28
Job’s method of continuous variation plot for the reaction of 4b salt with MO, [4b]=[MO]=0.27 mmol/L (Figure S6)………………………………………………...	S28

### 1-(4-bromophenyl)-2-ethyl-3,5-dimethylpyrazolium methanesulfonate (2a)

[Fig f7-turkjchem-45-6-1988]

**Figure f7-turkjchem-45-6-1988:**
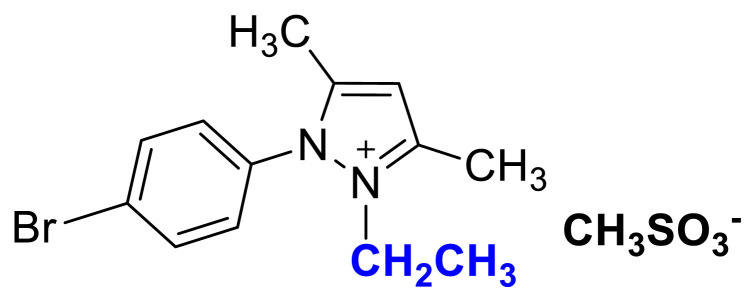


IR *ν*(cm^−1^): 3091, 2988, 1660, 1561, 1490, 1417, 1220, 1157, 1037, 772, 710. ^1^H NMR (DMSO-d_6_) *δ* (ppm): 1.10 (t, *J* = 7.2 Hz, 3H, −NCH_2_CH_3_), 2.18 (s, 3H, CH_3_), 2.35 (s, 3H, −CH_3_), 2.56 (s, 3H, −CH_3_), 4.13 (q, *J* = 7.2 Hz, 2H, −NCH_2_CH_3_), 6.83 (s, 1H, −CH), 7.73 (d, *J* = 8.7 Hz, 2H, Ph), 7.96 (d, *J* = 8.7 Hz, 2H, Ph). ^13^C NMR (DMSO-d_6_) *δ* (ppm): 11.81, 12.27, 14.30, 39.98, 43.04, 108.71, 126.65, 130.82, 131.66, 134.14, 147.66 and 148.0. Yield: (1.59 g) 85%, orange solid. M. p. 76.3 °C.

### 2-ethyl-1-(4-methoxyphenyl)-3,5-dimethylpyrazolium methanesulfonate (2b)

[Fig f8-turkjchem-45-6-1988]

**Figure f8-turkjchem-45-6-1988:**
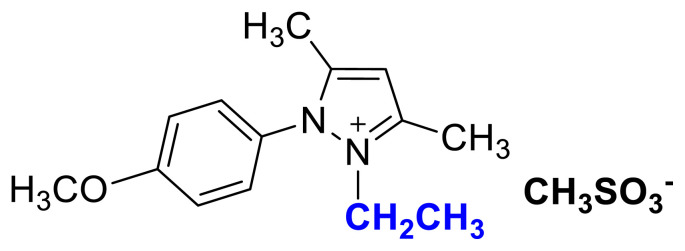


IR *ν*(cm^−1^): 3106, 2987, 1648, 1509, 1461, 1418, 1257, 1213, 1170, 1039, 773, 655.^1^H NMR (DMSO-d_6_) *δ* (ppm): 1.10 (t, *J* =7.2 Hz, 3H, −NCH_2_CH_3_), 2.16 (s, 3H, −CH_3_), 2.38 (s, 3H, −CH_3_), 2.55 (s, 3H, −CH_3_), 3.88 (s, 3H, PhOCH_3_), 4.10 (q, *J* = 7.2 Hz, 2H, −NCH_2_CH_3_), 6.80 (s, 1H, CH), 7.25 (d, *J* = 9.0 Hz, 2H, Ph,), 7.65 (d, *J* = 9.0 Hz, 2H, Ph).^13^C NMR (DMSO-d_6_) *δ* (ppm): 11.80, 12.28, 14.30, 40.13, 42.74, 56.30, 108.30, 116.06, 123.73, 131.01, 146.94, 147.93 and 162.17. Yield: (1.55 g) 95%, light brown viscous liquid.

### 2-ethyl-1-(4-nitrophenyl)-3,5-dimethylpyrazolium methanesulfonate (2c)

[Fig f9-turkjchem-45-6-1988]

**Figure f9-turkjchem-45-6-1988:**
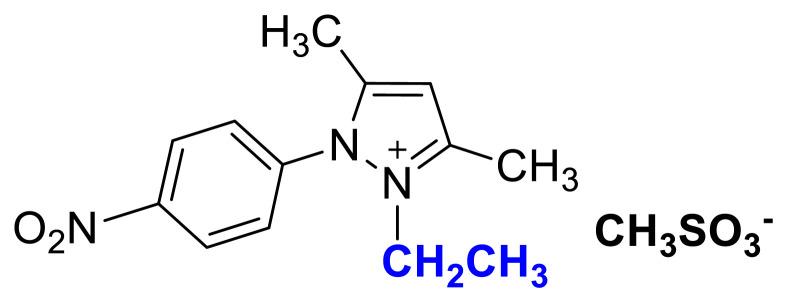


IR *ν* (cm^−1^): 3073, 2989, 2937, 1646, 1612, 1561, 1529, 1493, 1419, 1355, 1172, 1039, 877, 857, 772, 693. ^1^H NMR (CDCl_3_) *δ* (ppm): 1.16 (t, *J* = 7.2 Hz, 3H, −NCH_2_CH_3_), 2.23 (s, 3H, −CH_3_), 2.60 (s, 6H), 4.31 (q, *J* = 7.2 Hz, 2H, −NCH_2_CH_3_), 6.56 (s, 1H, CH), 8.13 (d, *J* = 8.8 Hz, 2H, Ph), 8.48 (d, *J* =8.8 Hz, 2H, Ph). ^13^C NMR (CDCl_3_) *δ* (ppm): 12.29, 12.44, 14.31, 39.38, 43.88, 109.18, 125.98, 131.23, 136.18, 147.0, 148.72 and 149.87. Yield: (1.42 g) 83%, yellow waxy solid.[Fig f10-turkjchem-45-6-1988][Fig f11-turkjchem-45-6-1988]

**Figure f10-turkjchem-45-6-1988:**
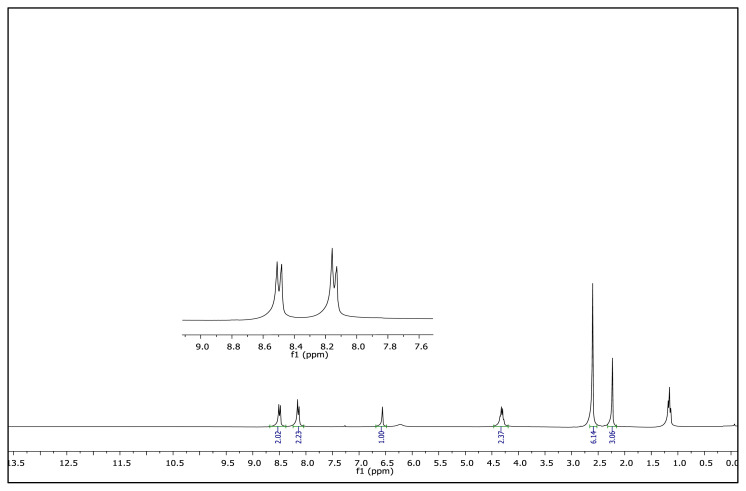
^1^H NMR spectrum of 2-ethyl-1-(4-nitrophenyl)-3,5-dimethylpyrazolium methanesulfonate (CDCl_3_)

**Figure f11-turkjchem-45-6-1988:**
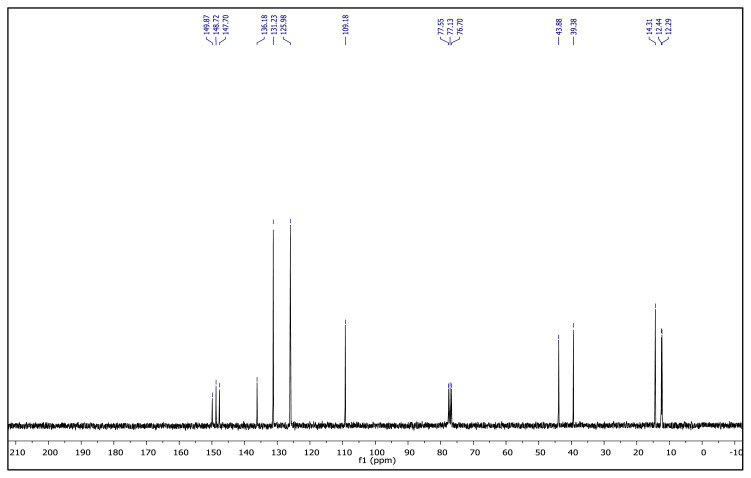
^13^C NMR spectrum of 2-ethyl-1-(4-nitrophenyl)-3,5-dimethylpyrazolium methanesulfonate (CDCl_3_)

### 1-(4-bromophenyl)-2-butyl-3,5-dimethylpyrazolium methanesulfonate (3a)

[Fig f12-turkjchem-45-6-1988]

**Figure f12-turkjchem-45-6-1988:**
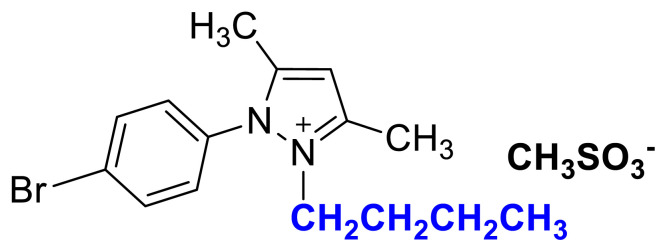


IR *ν*(cm^−1^): 3055, 2962, 1647, 1560, 1488, 1417, 1190, 1038, 767, 712. ^1^H NMR (DMSO-d_6_) *δ* (ppm): 0.7 (t, *J* = 7.3 Hz, 3H, −NCH_2_CH_2_CH_2_CH_3_), 1.10 (m, 2H, −NCH_2_CH_2_CH_2_CH_3_), 1.42 (m, 2H, −NCH_2_CH_2_CH_2_CH_3_), 2.19 (s, 3H, −CH_3_), 2.32 (s, 3H, −CH_3_), 2.56 (s, 3H, −CH_3_), 4.10 (t, *J* = 7.6 Hz, 2H, −NCH_2_CH_2_CH_2_CH_3_), 6.84 (s, 1H, CH), 7.74 (d, *J* = 8.6 Hz, 2H, Ph,), 7.97 (d, *J* = 8.6 Hz, 2H, Ph,). ^13^C NMR (DMSO-d_6_) *δ* (ppm): 12.06, 12.31, 13.49, 19.14, 30.45, 40.15, 47.24, 108.75, 126.51, 130.89, 131.70, 134.07, 148.09 and 148.15. Yield: (1.61 g) 80%, yellow viscous liquid.

### 2-butyl-1-(4-methoxyphenyl)-3,5-dimethylpyrazolium methanesulfonate (3b)

[Fig f13-turkjchem-45-6-1988]

**Figure f13-turkjchem-45-6-1988:**
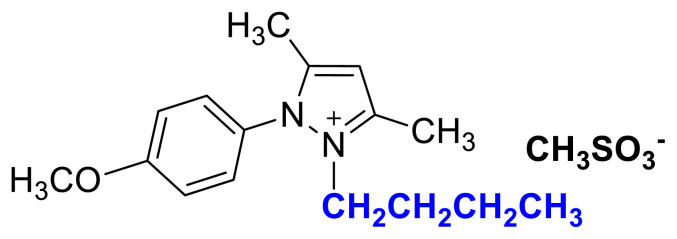


IR *ν*(cm^−1^): 3063, 2962, 1648, 1560, 1509, 1463, 1418, 1171, 1038, 767, 648. ^1^H NMR (DMSO-d_6_) *δ* (ppm): 0.69 (t, *J* = 7.3 Hz, 3H, −NCH_2_CH_2_CH_2_CH_3_), 1.10 (m, 2H, −NCH_2_CH_2_CH_2_CH_3_), 1.45 (m, 2H, −NCH_2_CH_2_CH_2_CH_3_), 2.17 (s, 3H, −CH_3_), 2.34 (s, 3H, −CH_3_), 2.55 (s, 3H, −CH_3_), 3.88 (s, 3H, PhOCH_3_), 4.07 (t, *J* = 7.6 Hz, 2H, −NCH_2_CH_2_CH_2_CH_3_), 6.81 (s, 1H, CH), 7.25 (d, *J* = 8.9 Hz, 2H, Ph), 7.66 (d, *J* = 8.9 Hz, 2H, Ph). ^13^C NMR (DMSO-d_6_) *δ* (ppm): 12.03, 12.31, 13.48, 19.17, 30.45, 40.12, 46.95, 56.31, 108.32, 115.98, 123.81, 131.04, 147.37, 147.99 and 162.14. Yield: (1.56 g) 88%, light brown viscous liquid.

### 2-butyl-1-(4-nitrophenyl)-3,5-dimethylpyrazolium methanesulfonate (3c)

[Fig f14-turkjchem-45-6-1988]

**Figure f14-turkjchem-45-6-1988:**
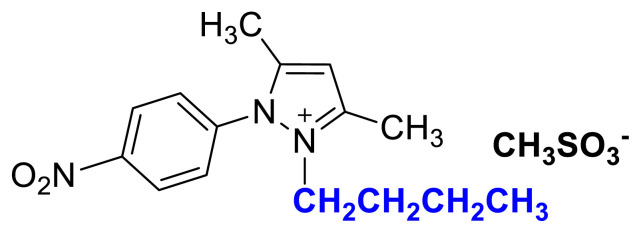


IR *ν*(cm^−1^): 3071, 2964, 2936, 2876, 1611, 1596, 1561, 1529, 1494, 1419, 1355, 1152, 1037, 856, 752, 694. ^1^H NMR (DMSO-d_6_) *δ* (ppm): 0.70 (t, *J* = 7.4 Hz, 3H, −NCH_2_CH_2_CH_2_CH_3_), 1.08 (m, 2H, −NCH_2_CH_2_CH_2_CH_3_), 1.43 (m, 2H,-NCH_2_CH_2_CH_2_CH_3_), 2.22 (s, 3H, −CH_3_), 2.40 (s, 3H, −CH_3_), 2.58 (s, 3H, −CH_3_), 4.14 (t, *J* = 7.4 Hz, 2H, −NCH_2_CH_2_CH_2_CH_3_), 6.90 (s, 1H, CH), 8.12 (d, *J* = 8.6 Hz, 2H, Ph), 8.55 (d, *J* = 8.6 Hz, 2H, Ph). ^13^C NMR (DMSO-d_6_) *δ* (ppm): 12.09, 12.32, 13.53, 19.11, 30.49, 40.10, 47.55, 47.42, 109.14, 126.14, 131.43, 136.60, 148.44, 148.96 and 149.93. Yield: (1.39 g) 73%, orange viscous liquid.[Fig f15-turkjchem-45-6-1988][Fig f16-turkjchem-45-6-1988]

**Figure f15-turkjchem-45-6-1988:**
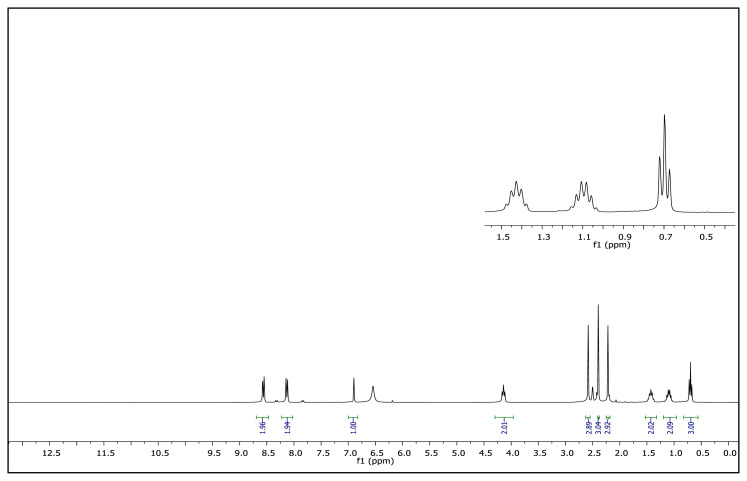
^1^H NMR spectrum of 2-butyl-1-(4-nitrophenyl)-3,5-dimethylpyrazolium methanesulfonate (DMSO-d_6_)

**Figure f16-turkjchem-45-6-1988:**
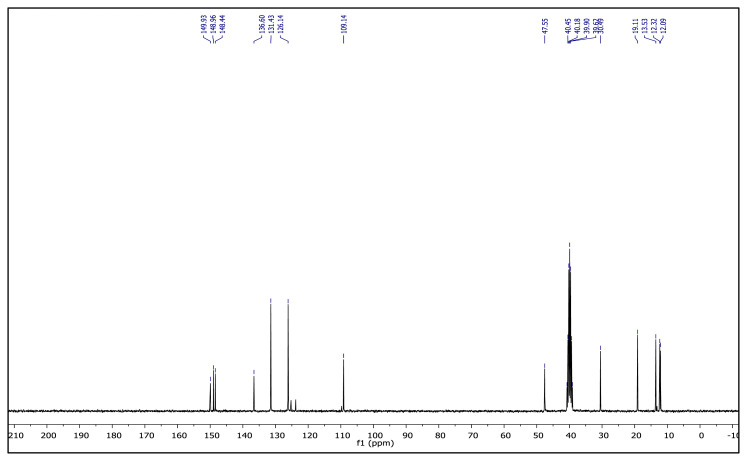
^13^C NMR spectrum of 2-butyl-1-(4-nitrophenyl)-3,5-dimethylpyrazolium methanesulfonate (DMSO-d_6_)

### 1-(4-bromophenyl)-2-ethyl-3,5-dimethylpyrazolium tetrafluoroborate (4a)

[Fig f17-turkjchem-45-6-1988]

**Figure f17-turkjchem-45-6-1988:**
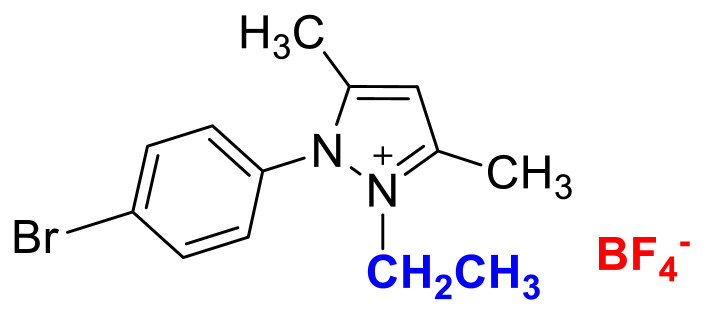


IR *ν*(cm^−1^): 3096, 2984, 1699, 1560, 1489, 1411, 1047, 1035, 1010, 864, 823, 652. ^1^H NMR (DMSO-d_6_) *δ* (ppm): 1.10 (t, *J* =7.2 Hz, 3H, −NCH_2_CH_3_), 2.17 (s, 3H, −CH_3_), 2.55 (s, 3H, −CH_3_), 4.11 (q, *J* = 7.2 Hz, 2H, −NCH_2_CH_3_), 6.81 (s, 1H, CH), 7.71 (d, *J* = 8.7 Hz, 2H, Ph), 7.97 (d, *J* = 8.7 Hz, 2H, Ph). ^13^C NMR (DMSO-d_6_) *δ* (ppm): 11.76, 12.24, 14.29, 43.0, 108.66, 126.70, 130.79, 131.60, 134.15, 147.62 and 148.02. ^19^F NMR (DMSO-d_6_) *δ* (ppm): −148.29, −148.23. Anal. calcd. for [C_13_H_16_BrN_2_][BF_4_]: C 42.55, H 4.39, N 7.63; found: C 42.44, H 4.41, N 7.61. HRMS (QTOF-ESI) m/z calcd. for C_13_H_16_BrN_2_: 279.0497; found: 279.0501. Yield: (1.72 g) 94%, beige solid. M. p. 164 °C.[Fig f18-turkjchem-45-6-1988][Fig f19-turkjchem-45-6-1988][Fig f20-turkjchem-45-6-1988][Fig f21-turkjchem-45-6-1988][Fig f22-turkjchem-45-6-1988]

**Figure f18-turkjchem-45-6-1988:**
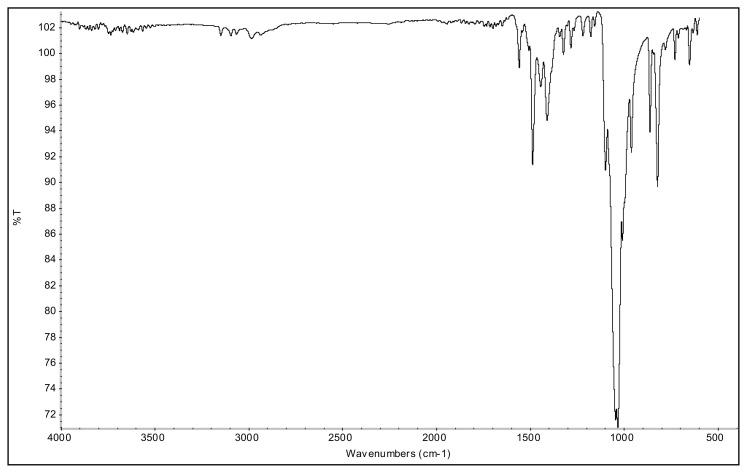
IR spectrum of 1-(4-bromophenyl)-2-ethyl-3,5-dimethylpyrazolium tetrafluoroborate

**Figure f19-turkjchem-45-6-1988:**
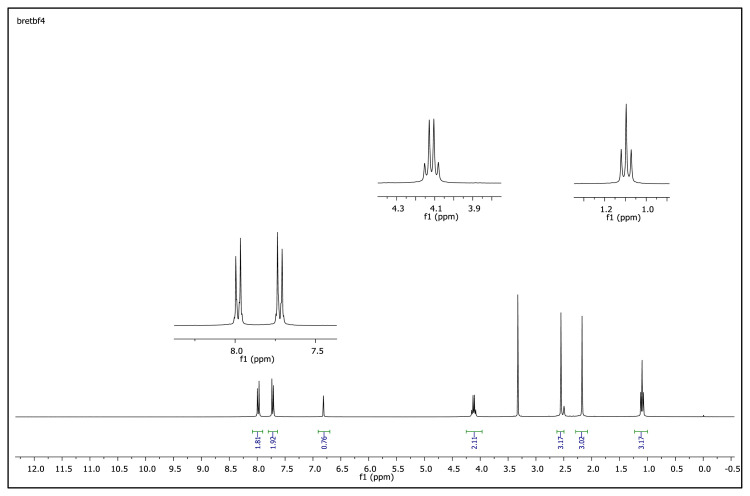
^1^H NMR spectrum of 1-(4-bromophenyl)-2-ethyl-3,5-dimethylpyrazolium tetrafluoroborate (DMSO-d_6_)

**Figure f20-turkjchem-45-6-1988:**
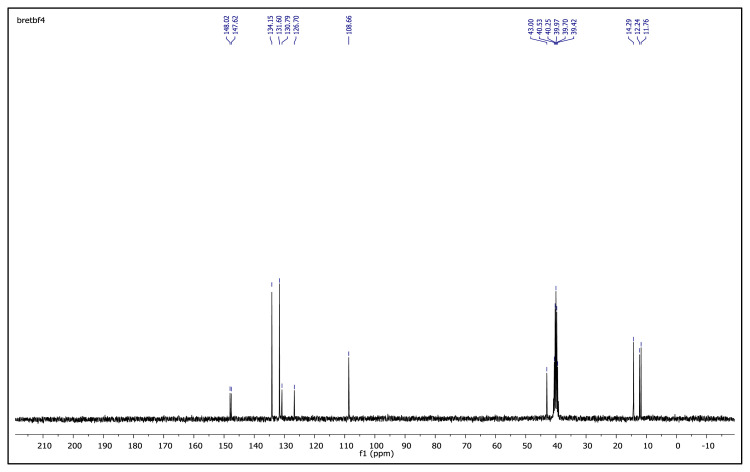
^13^C NMR spectrum of 1-(4-bromophenyl)-2-ethyl-3,5-dimethylpyrazolium tetrafluoroborate (DMSO-d_6_)

**Figure f21-turkjchem-45-6-1988:**
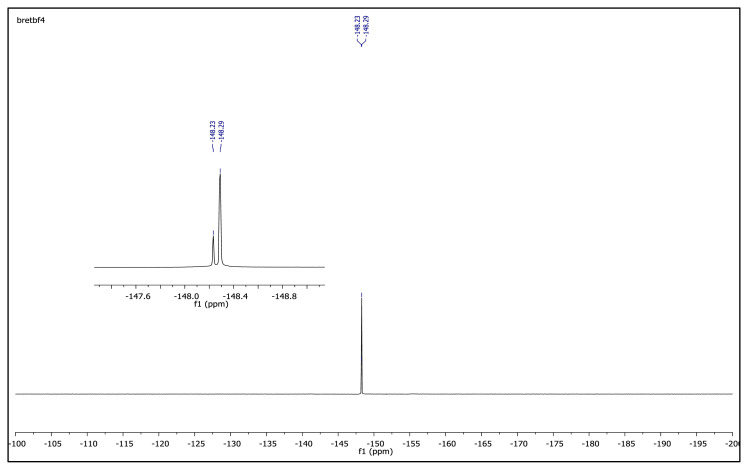
^19^F NMR spectrum of 1-(4-bromophenyl)-2-ethyl-3,5-dimethylpyrazolium tetrafluoroborate (DMSO-d_6_)

**Figure f22-turkjchem-45-6-1988:**
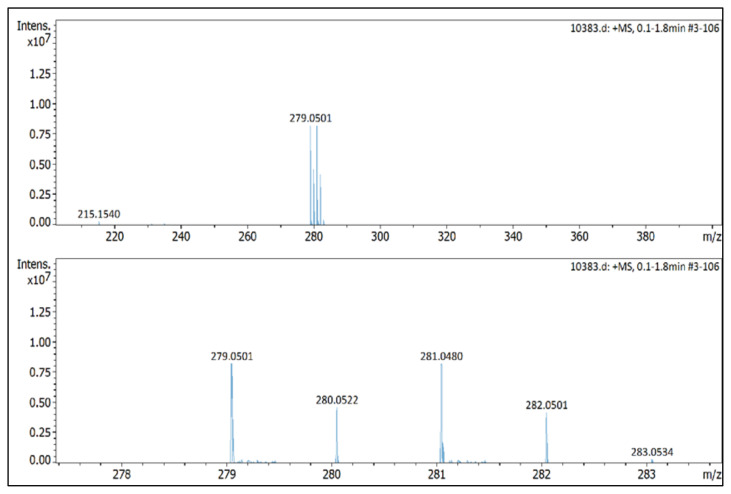
HRMS spectrum of 1-(4-bromophenyl)-2-ethyl-3,5-dimethylpyrazolium tetrafluoroborate

### 2-ethyl-1-(4-methoxyphenyl)-3,5-dimethylpyrazolium tetrafluoroborate (4b)

[Fig f23-turkjchem-45-6-1988]

**Figure f23-turkjchem-45-6-1988:**
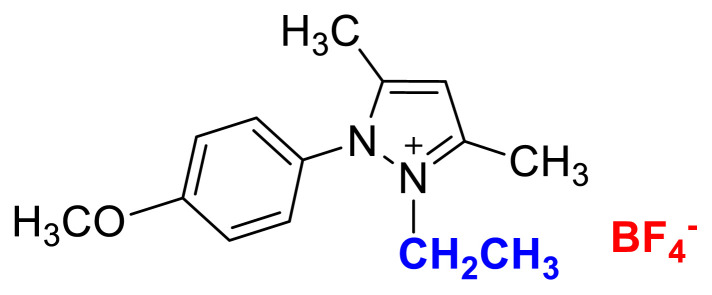


IR *ν*(cm^−1^): 3070, 2988, 2942, 2848, 1604, 1560, 1510, 1482, 1251, 1050, 1037, 962, 857, 828. ^1^H NMR (DMSO-d_6_) *δ* (ppm): 1.11 (t, *J* = 7.2 Hz, 3H, −NCH_2_CH_3_), 2.16 (s, 3H, −CH_3_), 2.55 (s, 3H, −CH_3_), 3.89 (s, 3H, PhOCH_3_), 4.08 (q, *J* = 7.2 Hz, 2H, −NCH_2_CH_3_), 6.78 (s, 1H, CH), 7.25 (d, *J* = 9.0 Hz, 2H, Ph), 7.65 (d, *J* = 9.0 Hz, 2H, Ph). ^13^C NMR (DMSO-d_6_) *δ* (ppm): 11.72, 12.24, 14.27, 42.68, 56.25, 108.25, 116.05, 123.71, 130.97, 146.91, 147.96 and 162.19. ^19^F NMR (DMSO-d_6_) *δ* (ppm): −148.30, −148.25. Anal. calcd. for [C_14_H_19_N_2_O][BF_4_]: C 52.86, H 6.02, N 8.81; found: C 52.71, H 6.03, N 8.78. HRMS (QTOF-ESI) m/z calcd. for C_14_H_19_N_2_O: 231.1497; found: 231.1510. Yield: (1.44 g) 90.5%, beige solid. M. p. 140.0 °C.[Fig f24-turkjchem-45-6-1988][Fig f25-turkjchem-45-6-1988][Fig f26-turkjchem-45-6-1988][Fig f27-turkjchem-45-6-1988][Fig f28-turkjchem-45-6-1988]

**Figure f24-turkjchem-45-6-1988:**
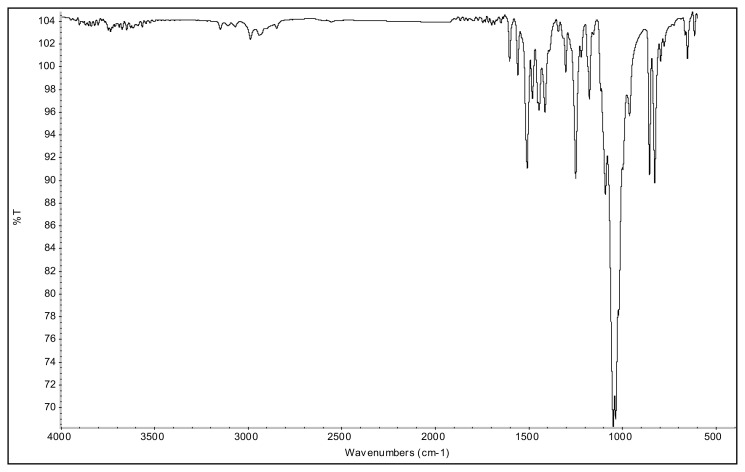
IR spectrum of 2-ethyl-1-(4-methoxyphenyl)-3,5-dimethylpyrazolium tetrafluoroborate

**Figure f25-turkjchem-45-6-1988:**
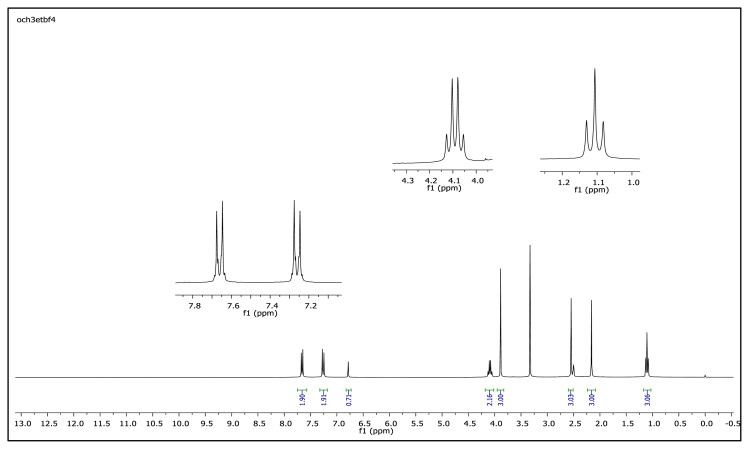
^1^H NMR spectrum of 2-ethyl-1-(4-methoxyphenyl)-3,5-dimethylpyrazolium tetrafluoroborate (DMSO-d_6_)

**Figure f26-turkjchem-45-6-1988:**
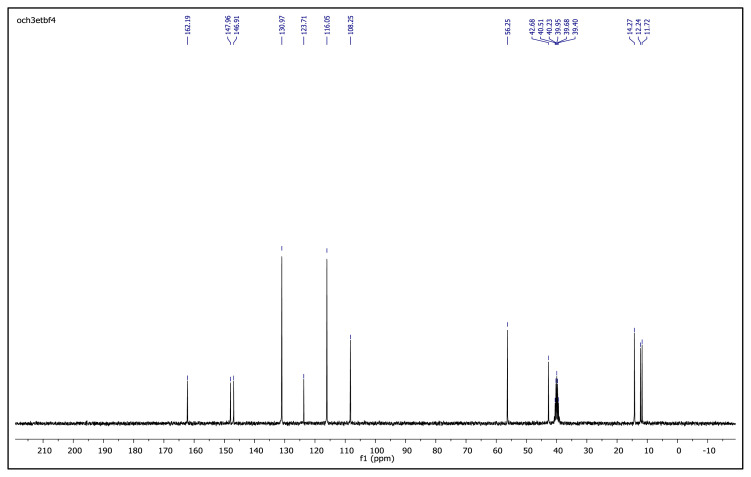
^13^C NMR spectrum of 2-ethyl-1-(4-methoxyphenyl)-3,5-dimethylpyrazolium tetrafluoroborate (DMSO-d_6_)

**Figure f27-turkjchem-45-6-1988:**
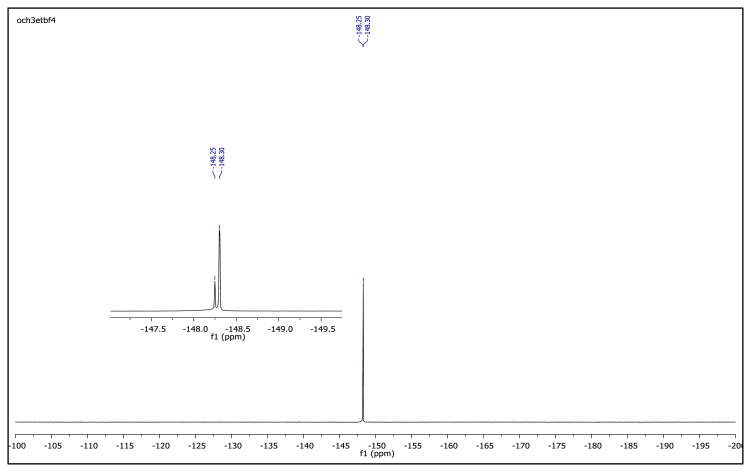
^19^F NMR spectrum of 2-ethyl-1-(4-methoxyphenyl)-3,5-dimethylpyrazolium tetrafluoroborate (DMSO-d_6_)

**Figure f28-turkjchem-45-6-1988:**
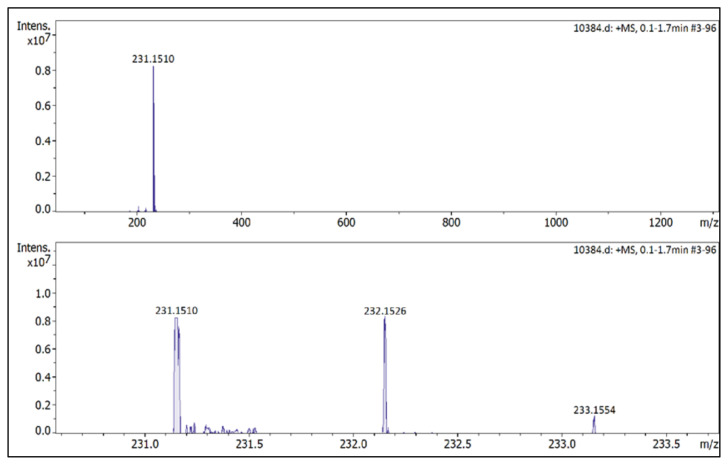
HRMS spectrum of 2-ethyl-1-(4-methoxyphenyl)-3,5-dimethylpyrazolium tetrafluoroborate

### 2-ethyl-1-(4-nitrophenyl)-3,5-dimethylpyrazolium tetrafluoroborate (4c)

[Fig f29-turkjchem-45-6-1988]

**Figure f29-turkjchem-45-6-1988:**
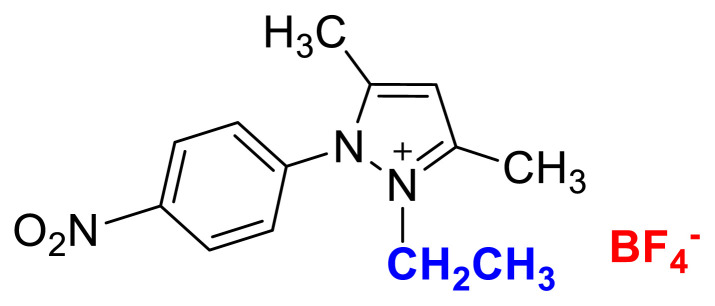


IR *ν*(cm^−1^): 3087, 2995, 2945, 1615, 1559, 1527, 1492, 1357, 1047, 1033, 968, 857, 750. ^1^H NMR (DMSO-d_6_,) *δ* (ppm): 1.11 (t, *J* = 7.2 Hz, 3H, −NCH_2_CH_3_), 2.22 (s, 3H, −CH_3_), 2.59 (s, 3H, −CH_3_), 4.16 (q, *J* = 7.2 Hz, 2H, −NCH_2_CH_3_), 6.88 (s, 1H, CH), 8.09 (d, *J* = 8.9 Hz, 2H, Ph), 8.57 (d, *J* = 8.9 Hz, 2H, Ph). ^13^C NMR (DMSO-d_6_) *δ* (ppm): 11.78, 12.22, 14.25, 43.28, 109.07, 126.22, 131.39, 136.51, 148.29, 148.42 and 150.05. ^19^F NMR (DMSO-d_6_) *δ* (ppm): −148.29, −148.24. Anal. calcd. for [C_13_H_16_N_3_O_2_][BF_4_]: C 46.88, H 4.84, N 12.62; found: C 46.75, H 4.86, N 12.58. HRMS (QTOF-ESI) m/z calcd. for C_13_H_16_N_3_O_2_: 246.1243; found: 246.1247. Yield: (1.46 g) 87.7%, yellow solid. M. p. 198.0 °C.[Fig f30-turkjchem-45-6-1988][Fig f31-turkjchem-45-6-1988][Fig f32-turkjchem-45-6-1988][Fig f33-turkjchem-45-6-1988][Fig f34-turkjchem-45-6-1988]

**Figure f30-turkjchem-45-6-1988:**
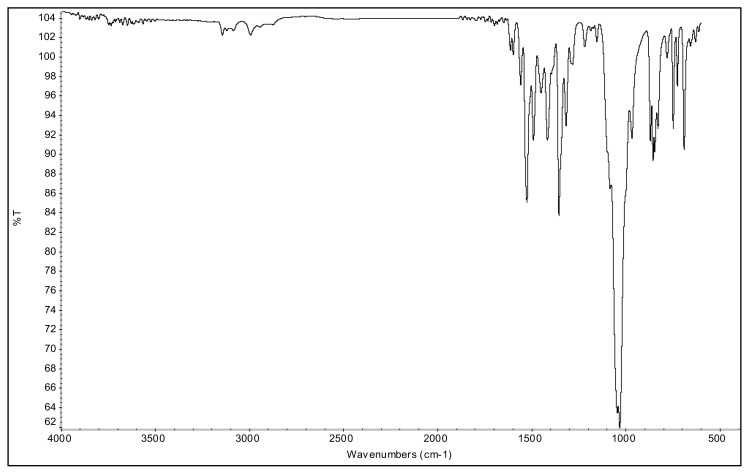
IR spectrum of 2-ethyl-1-(4-nitrophenyl)-3,5-dimethylpyrazolium tetrafluoroborate

**Figure f31-turkjchem-45-6-1988:**
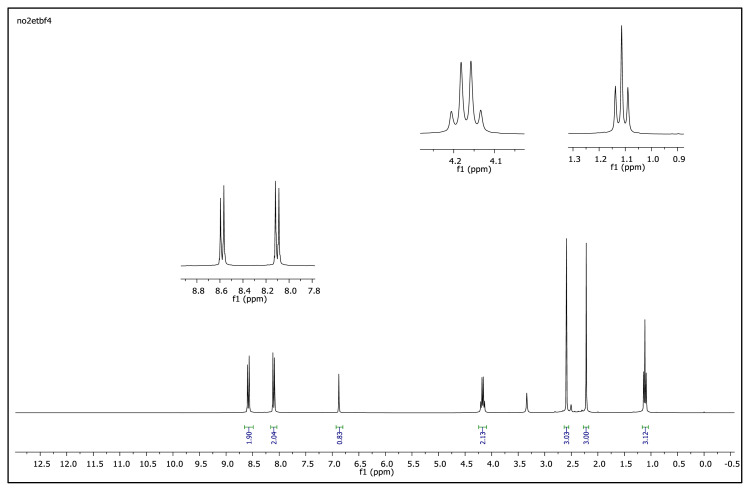
^1^H NMR spectrum of 2-ethyl-1-(4-nitrophenyl)-3,5-dimethylpyrazolium tetrafluoroborate (DMSO-d_6_)

**Figure f32-turkjchem-45-6-1988:**
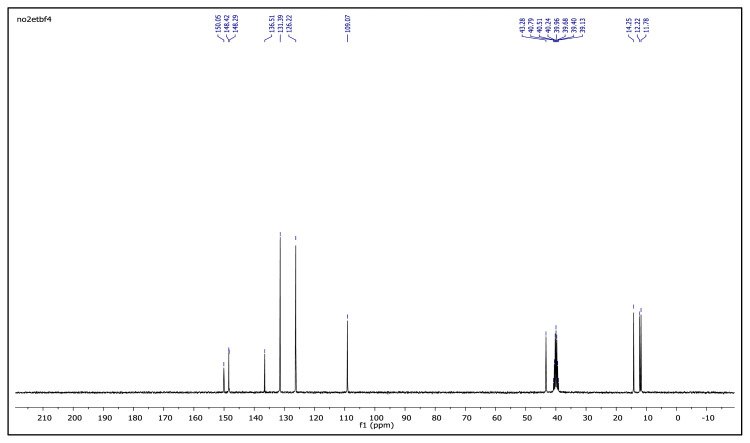
^13^C NMR spectrum of 2-ethyl-1-(4-nitrophenyl)-3,5-dimethylpyrazolium tetrafluoroborate (DMSO-d_6_)

**Figure f33-turkjchem-45-6-1988:**
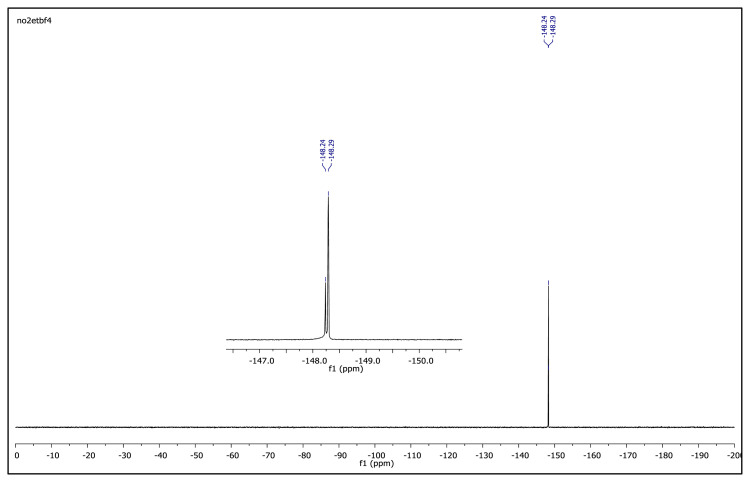
^19^F NMR spectrum of 2-ethyl-1-(4-nitrophenyl)-3,5-dimethylpyrazolium tetrafluoroborate (DMSO-d_6_)

**Figure f34-turkjchem-45-6-1988:**
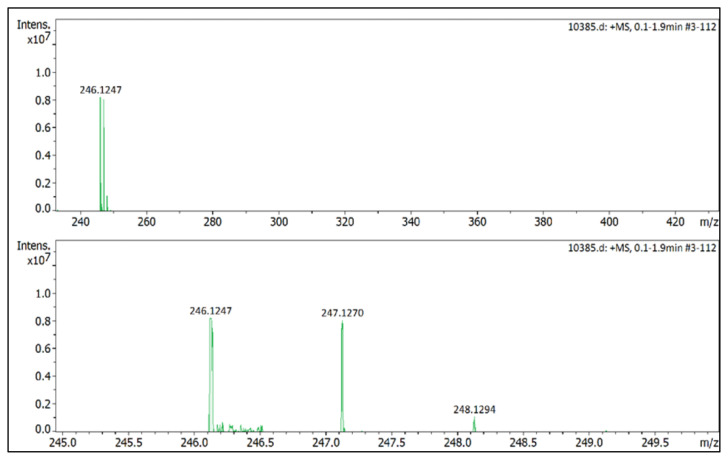
HRMS spectrum of 2-ethyl-1-(4-nitrophenyl)-3,5-dimethylpyrazolium tetrafluoroborate

### 1-(4-bromophenyl)-2-butyl-3,5-dimethylpyrazolium tetrafluoroborate (5a)

[Fig f35-turkjchem-45-6-1988]

**Figure f35-turkjchem-45-6-1988:**
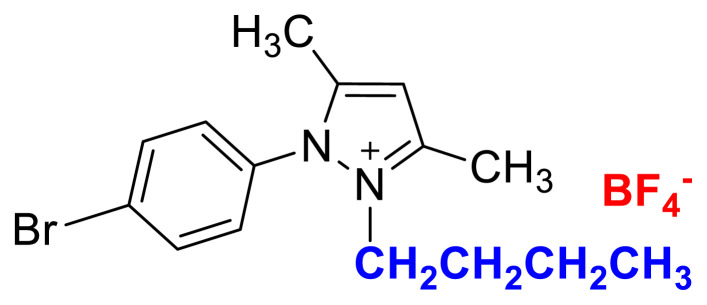


IR *ν*(cm^−1^): 3103, 2966, 2869, 1650, 1561, 1511, 1487, 1418, 1045, 1033, 1009, 855, 820, 679. ^1^H NMR (DMSO-d_6_) *δ* (ppm): 0.71 (t, *J* = 7.3 Hz, 3H, −NCH_2_CH_2_CH_2_CH_3_), 1.10 (m, 2H, −NCH_2_CH_2_CH_2_CH_3_), 1.44 (m, 2H, −NCH_2_CH_2_CH_2_CH_3_), 2.19 (s, 3H, −CH_3_), 2.56 (s, 3H, −CH_3_), 4.09 (t, *J* = 7.6 Hz, 2H, −NCH_2_CH_2_CH_2_CH_3_), 6.83 (s, 1H, CH), 7.72 (d, *J* = 8.6 Hz, 2H, Ph), 7.98 (d, *J* =8.6 Hz, 2H, Ph). ^13^C NMR (DMSO-d_6_) *δ* (ppm): 11.99, 12.29, 13.50, 19.12, 30.49, 47.17, 108.65, 126.60, 130.85, 131.59, 134.09, 148.06, 148.14. ^19^F NMR (DMSO-d_6_) *δ* (ppm): −148.31, −148.26. Anal. calcd for [C_15_H_20_BrN_2_][BF_4_]: C 45.61, H 5.10, N 7.09; found: C 45.50, H 5.12, N 7.07. HRMS (QTOF-ESI) m/z calcd. for C_15_H_20_BrN_2_: 307.0810; found: 307.0823. Yield: (1.84 g) 93.2%, white solid. M. p. 92.0 °C.[Fig f36-turkjchem-45-6-1988][Fig f37-turkjchem-45-6-1988][Fig f38-turkjchem-45-6-1988][Fig f39-turkjchem-45-6-1988][Fig f40-turkjchem-45-6-1988]

**Figure f36-turkjchem-45-6-1988:**
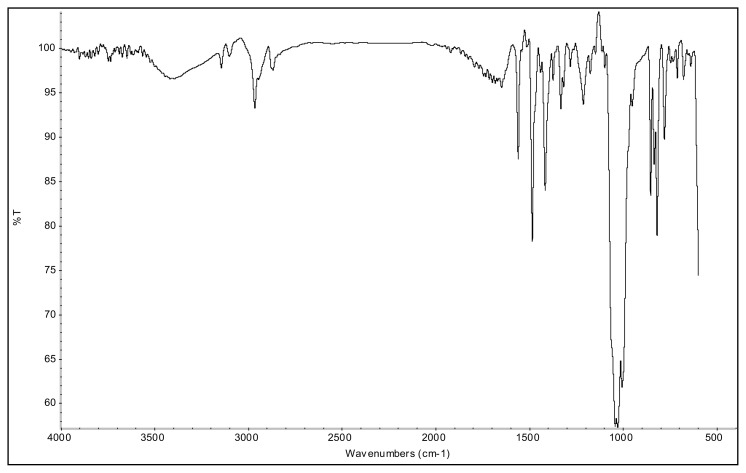
IR spectrum of 1-(4-bromophenyl)-2-butyl-3,5-dimethylpyrazolium tetrafluoroborate

**Figure f37-turkjchem-45-6-1988:**
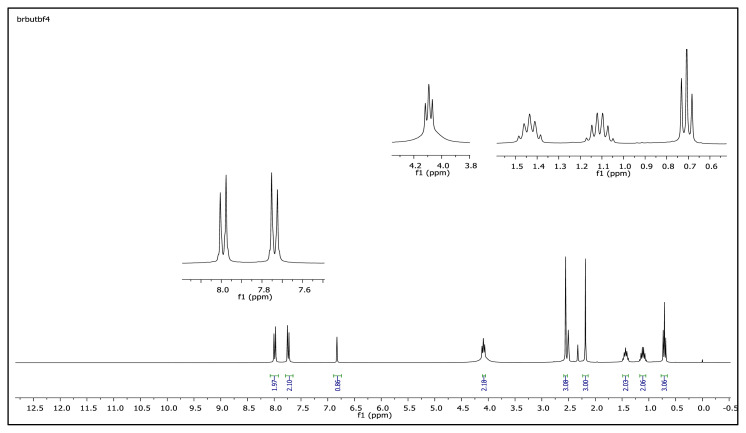
^1^H NMR spectrum of 1-(4-bromophenyl)-2-butyl-3,5-dimethylpyrazolium tetrafluoroborate (DMSO-d_6_)

**Figure f38-turkjchem-45-6-1988:**
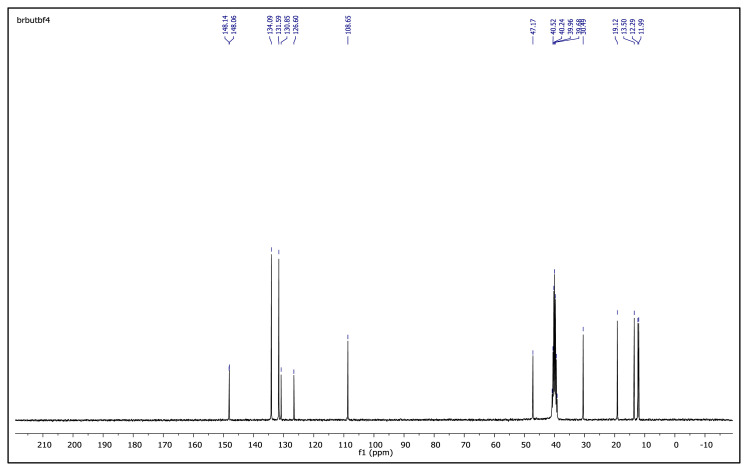
^13^C NMR spectrum of 1-(4-bromophenyl)-2-butyl-3,5-dimethylpyrazolium tetrafluoroborate (DMSO-d_6_)

**Figure f39-turkjchem-45-6-1988:**
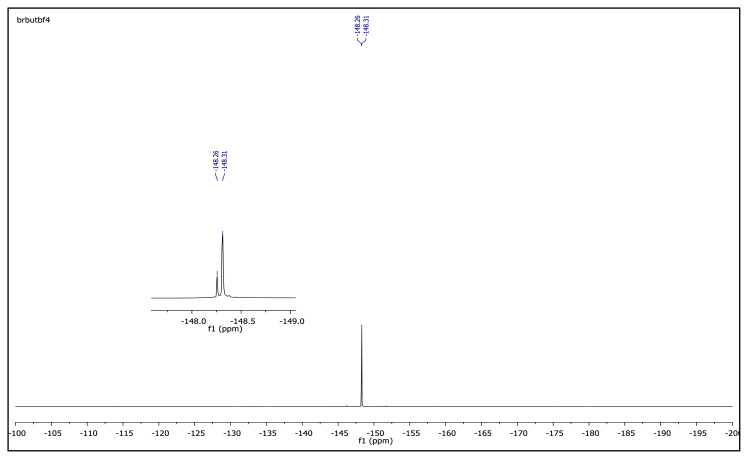
^19^F NMR spectrum of 1-(4-bromophenyl)-2-butyl-3,5-dimethylpyrazolium tetrafluoroborate (DMSO-d_6_)

**Figure f40-turkjchem-45-6-1988:**
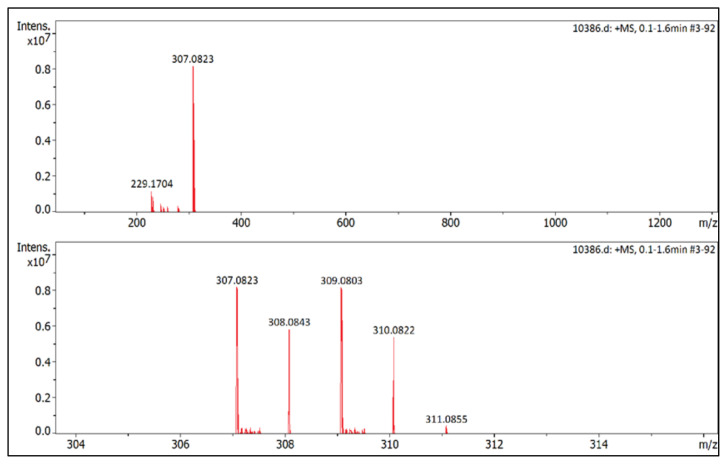
HRMS spectrum of 1-(4-bromophenyl)-2-butyl-3,5-dimethylpyrazolium tetrafluoroborate

#### 2-butyl-1-(4-methoxyphenyl)-3,5-dimethylpyrazolium tetrafluoroborate (5b)

[Fig f41-turkjchem-45-6-1988]

**Figure f41-turkjchem-45-6-1988:**
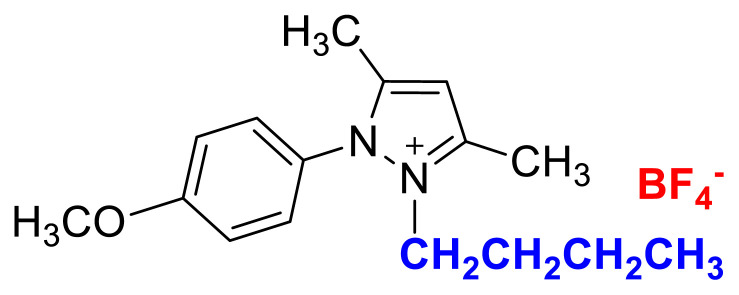


IR *ν*(cm^−1^): 3082, 2961, 2878, 1602, 1561, 1514, 1474, 1261, 1093, 1049, 1035, 854, 833. ^1^H NMR (DMSO-d_6_,) *δ* (ppm): 0.71 (t, *J* = 7.4 Hz, 3H,-NCH_2_CH_2_CH_2_CH_3_), 1.10 (m, 2H, −NCH_2_CH_2_CH_2_CH_3_), 1.45 (m, 2H, −NCH_2_CH_2_CH_2_CH_3_), 2.17 (s, 3H, −CH_3_), 2.54 (s, 3H, −CH_3_), 3.89 (s, 3H, PhOCH_3_), 4.06 (t, *J* = 7.6 Hz, 2H, −NCH_2_CH_2_CH_2_CH_3_), 6.79 (s, 1H, CH), 7.24 (d, *J* = 9.0 Hz, 2H, Ph), 7.65 (d, *J* = 9.0 Hz, 2H, Ph). ^13^C NMR (DMSO-d_6_) *δ* (ppm): 11.38, 12.72, 12.91, 18.58, 29.90, 46.30, 55.68, 107.64, 115.39, 123.18, 130.39, 146.69, 147.46, 161.58. ^19^F NMR (DMSO-d_6_) *δ* (ppm): −148.28, −148.23. Anal. calcd for [C_16_H_23_N_2_O][BF_4_]: C 55.51, H 6.70, N 8.09; found: C 55.37, H 6.71, N 8.07. HRMS (QTOF-ESI) m/z calcd. for C_16_H_23_N_2_O: 259.1810; found: 259.1824. Yield: (1.55) 89.6%, beige solid. M. p. 89.0 °C.[Fig f42-turkjchem-45-6-1988][Fig f43-turkjchem-45-6-1988][Fig f44-turkjchem-45-6-1988][Fig f45-turkjchem-45-6-1988][Fig f46-turkjchem-45-6-1988]

**Figure f42-turkjchem-45-6-1988:**
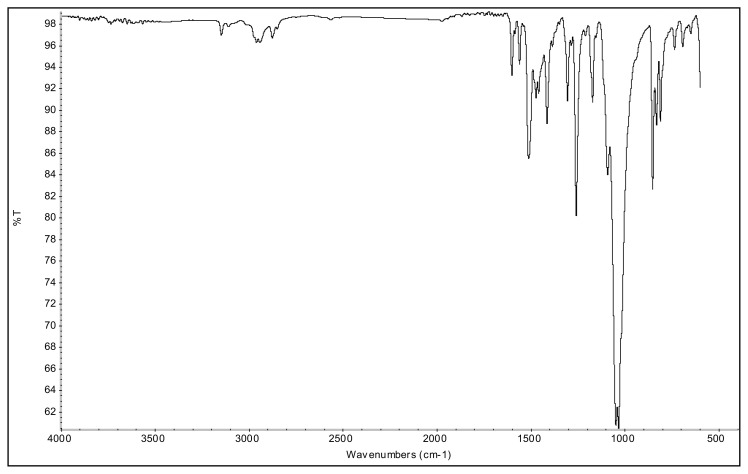
IR spectrum of 2-butyl-1-(4-methoxyphenyl)-3,5-dimethylpyrazolium tetrafluoroborate

**Figure f43-turkjchem-45-6-1988:**
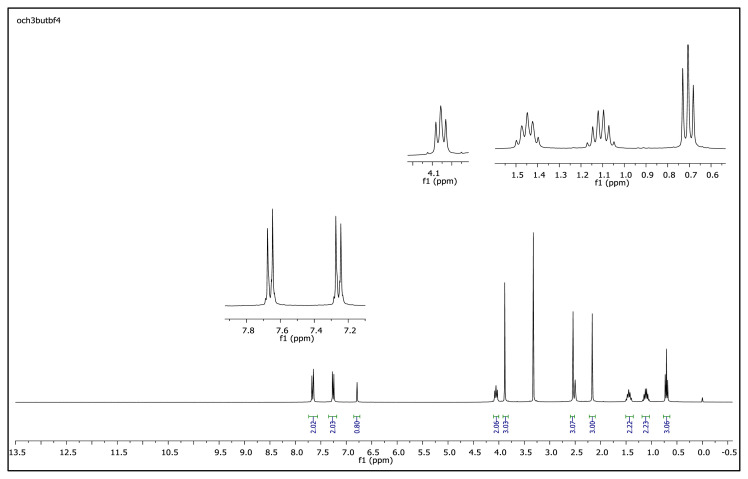
^1^H NMR spectrum of 2-butyl-1-(4-methoxyphenyl)-3,5-dimethylpyrazolium tetrafluoroborate (DMSO-d_6_)

**Figure f44-turkjchem-45-6-1988:**
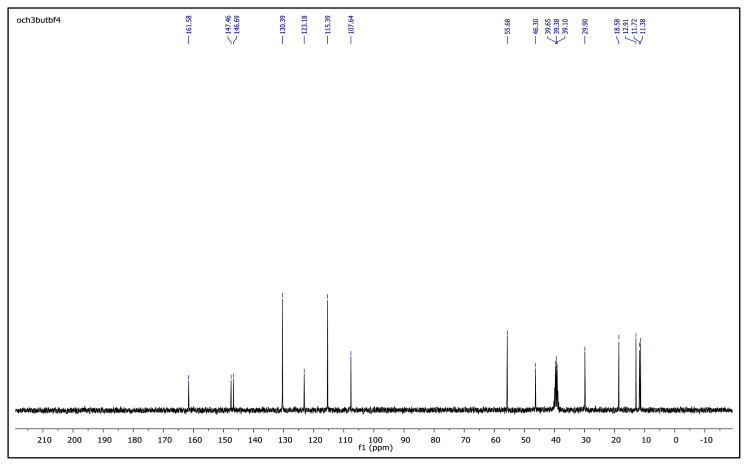
^13^C NMR spectrum of 2-butyl-1-(4-methoxyphenyl)-3,5-dimethylpyrazolium tetrafluoroborate (DMSO-d_6_)

**Figure f45-turkjchem-45-6-1988:**
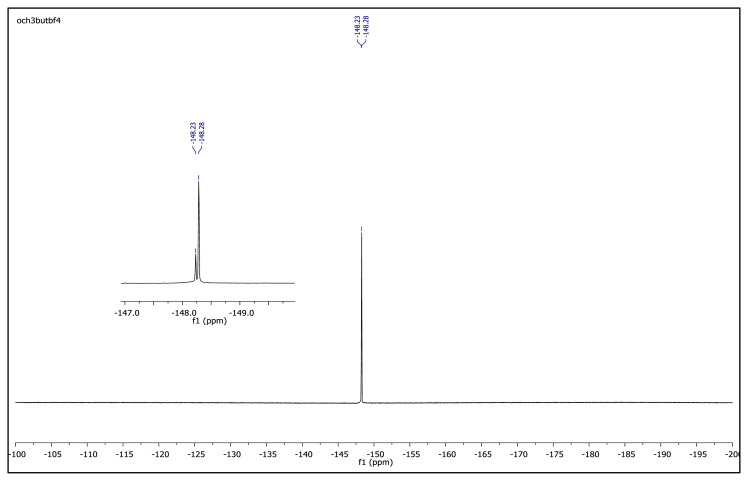
^19^F NMR spectrum of 2-butyl-1-(4-methoxyphenyl)-3,5-dimethylpyrazolium tetrafluoroborate (DMSO-d_6_)

**Figure f46-turkjchem-45-6-1988:**
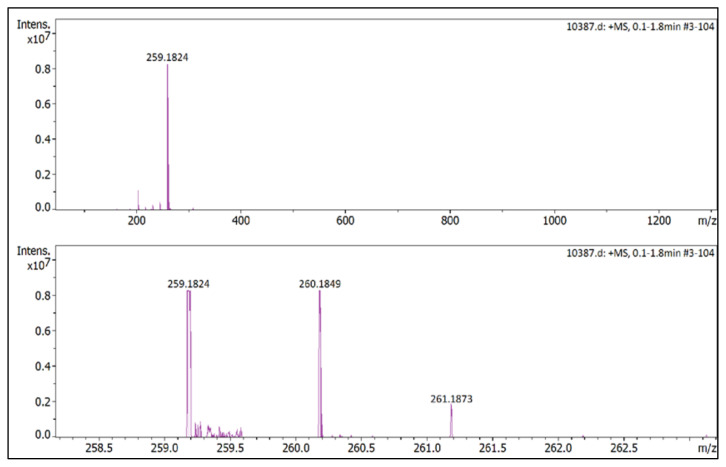
HRMS spectrum of 2-butyl-1-(4-methoxyphenyl)-3,5-dimethylpyrazolium tetrafluoroborate

### 2-butyl-1-(4-nitrophenyl)-3,5-dimethylpyrazolium tetrafluoroborate (5c)

[Fig f47-turkjchem-45-6-1988]

**Figure f47-turkjchem-45-6-1988:**
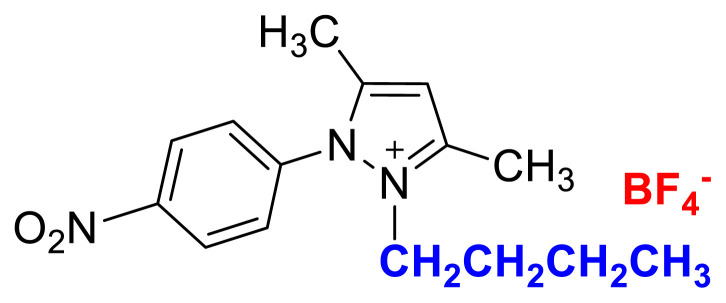


IR *ν*(cm^−1^): 3078, 2967, 2879, 1614, 1561, 1537, 1493, 1355, 1040, 1033, 826, 750, 690. ^1^H NMR (DMSO-d_6_) *δ* (ppm): 0.71 (t, *J* = 7.4 Hz, 3H,-NCH_2_CH_2_CH_2_CH_3_), 1.09 (m, 2H, −NCH_2_CH_2_CH_2_CH_3_), 1.43 (m, 2H, −NCH_2_CH_2_CH_2_CH_3_), 2.21 (s, 3H, −CH_3_), 2.58 (s, 3H, −CH_3_), 4.13 (t, *J* = 7.7 Hz, 2H,-NCH_2_CH_2_CH_2_CH_3_), 6.88 (s, 1H, CH), 8.08 (d, *J* = 8.9 Hz, 2H, Ph), 8.57 (d, *J* = 8.9 Hz, 2H, Ph). ^13^C NMR (DMSO-d_6_) *δ* (ppm): 12.02, 12.28, 13.54, 19.11, 30.53, 47.48, 109.06, 126.18, 131.34, 136.57, 148.46, 148.87, 149.98. ^19^F NMR (DMSO-d_6_) *δ* (ppm): −148.27, −148.21. Anal. calcd for [C_15_H_20_N_3_O_2_][BF_4_]: C 49.89, H 5.58, N 11.64; found: C 49.76, H 5.60, N 11.61. HRMS (QTOF-ESI) m/z calcd. for C_15_H_20_N_3_O_2_: 274.1556; found: 274.1568. Yield: (1.48 g) 82.0%, yellow solid. M. p. 93.0 °C.[Fig f48-turkjchem-45-6-1988][Fig f49-turkjchem-45-6-1988][Fig f50-turkjchem-45-6-1988][Fig f51-turkjchem-45-6-1988][Fig f52-turkjchem-45-6-1988]

**Figure f48-turkjchem-45-6-1988:**
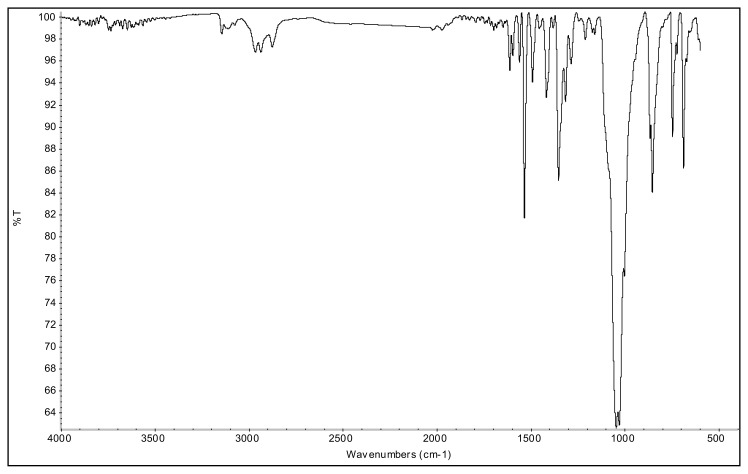
IR spectrum of 2-butyl-1-(4-nitrophenyl)-3,5-dimethylpyrazolium tetrafluoroborate

**Figure f49-turkjchem-45-6-1988:**
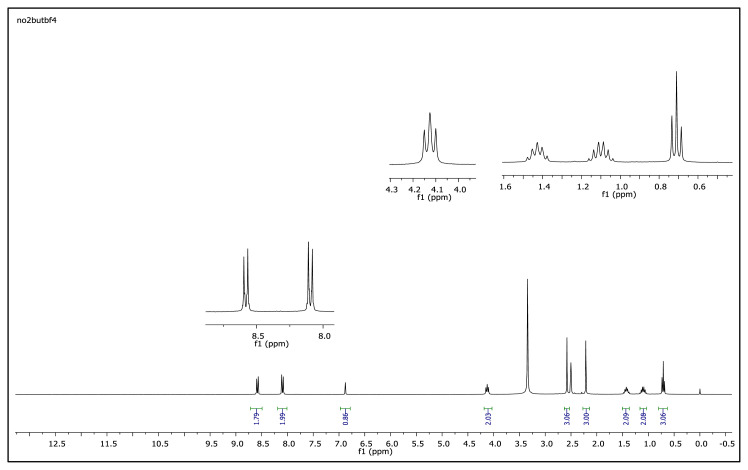
^1^H NMR spectrum of 2-butyl-1-(4-nitrophenyl)-3,5-dimethylpyrazolium tetrafluoroborate (DMSO-d_6_)

**Figure f50-turkjchem-45-6-1988:**
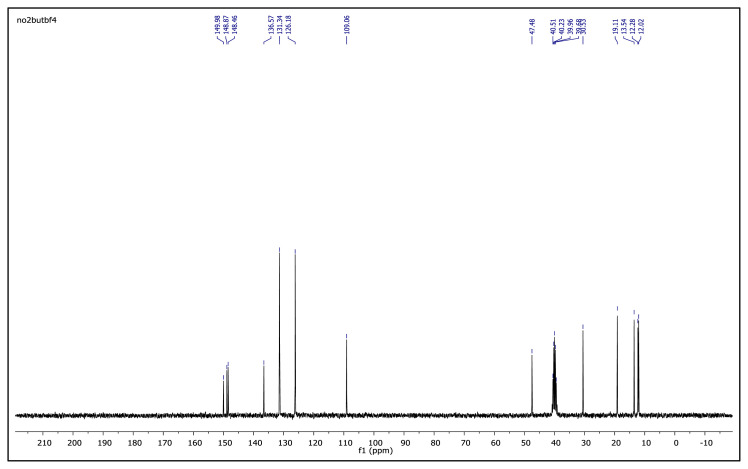
^13^C NMR spectrum of 2-butyl-1-(4-nitrophenyl)-3,5-dimethylpyrazolium tetrafluoroborate (DMSO-d_6_)

**Figure f51-turkjchem-45-6-1988:**
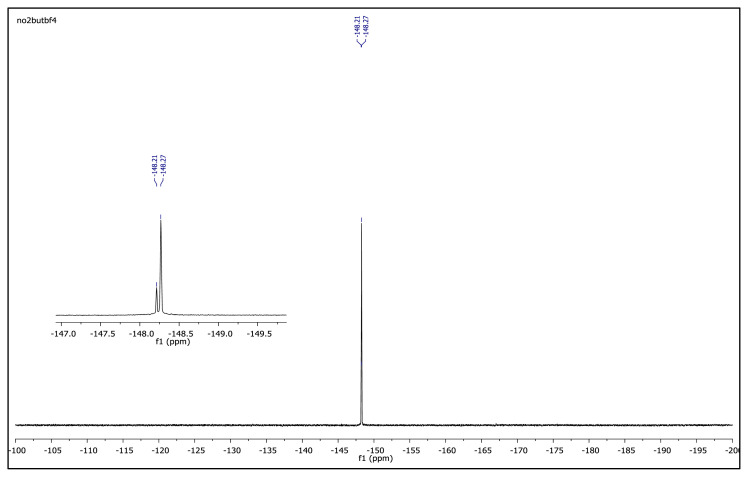
^19^F NMR spectrum of 2-butyl-1-(4-nitrophenyl)-3,5-dimethylpyrazolium tetrafluoroborate (DMSO-d_6_)

**Figure f52-turkjchem-45-6-1988:**
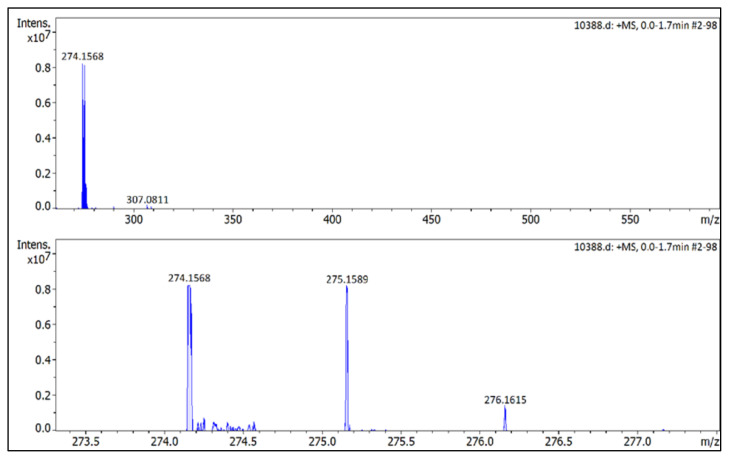
HRMS spectrum of 2-butyl-1-(4-nitrophenyl)-3,5-dimethylpyrazolium tetrafluoroborate

### Calibration curve of methyl orange

The stock solution (0.54 mmol/L) was prepared by dissolving an appropriate amount of methyl orange in ultrapure water and diluted to acquire MO solutions with different concentrations (0.1–0.001 mmol/L). Their absorbances were measured at the maximum absorption wavelength of MO (464 nm).

Figure S1 Calibration curve of methyl orange (0.1–0.001 mmol/L).

Figure S2 The main structures of MO in solutions (A) anionic form with pH above 3.8 (yellow) (B) zwitterionic form with pH below 3.8 (red).

**Table S1:** The removal efficiencies of TAAILs with 0.02, 0.04, and 0.1 MO/TAAIL molar ratios (Initial concentration of MO: 0.54 mmol/L).

TAAIL	Entry	Quantity of TAAIL/mg (mmol)	MO/TAAIL mol ratio	Removal Efficiency(%) *	D
4a	1	30 (0.082)	0.02	99.4	153
	2	15.4 (0.042)	0.04	99.2	128
	3	6.2 (0.017)	0.1	98.8	79
4b	1	26 (0.082)	0.02	99.2	124
	2	13 (0.041)	0.04	99.1	106
	3	5.2 (0.016)	0.1	98.4	61
4c	1	27 (0.081)	0.02	97.9	46
	2	13.6 (0.041)	0.04	97.3	37
	3	5 (0.015)	0.1	94.4	17
5a	1	32 (0.081)	0.02	99.7	340
	2	16.3 (0.041)	0.04	99.3	144
	3	6.7 (0.017)	0.1	99.1	113
5b	1	28.6 (0.083)	0.02	99.7	320
	2	14.3 (0.041)	0.04	99.6	272
	3	5.8 (0.017)	0.1	99.4	169
5c	1	29.5 (0.082)	0.02	98.4	60
	2	14.8 (0.041)	0.04	98.3	58
	3	5.9 (0.016)	0.1	98.2	56

* All experiments were conducted two times, and the data presented are an average of the obtained values.

**Table S2:** The removal efficiencies of TAAILs with 0.2, 0.4, and 1.0 MO/TAAIL molar ratios (Initial concentration of MO: 0.54 mmol/L).

TAAIL	Entry	Quantity of TAAIL/mg (mmol)	MO/TAAIL mol ratio	Removal efficiency (%) *	D
4a	1	3.4 (9.26 × 10 ^−3^)	0.2	97.7	43
	2	1.6 (4.36 × 10 ^−3^)	0.4	96.4	27
	3	0.6 (1.64 × 10 ^−3^)	1	89.5	9
4b	1	2.6 (8.17 × 10 ^−3^)	0.2	97.7	42
	2	1.3 (4.09 × 10 ^−3^)	0.4	95.8	23
	3	0.5 (1.57 × 10 ^−3^)	1	90.9	10
4c	1	2.8 (8.41 × 10 ^−3^)	0.2	91.5	11
	2	1.5 (4.50 × 10 ^−3^)	0.4	87.5	7
	3	0.5 (1.50 × 10 ^−3^)	1	74.5	3
5a	1	3.3 (8.35 × 10 ^−3^)	0.2	98.3	59
	2	1.7 (4.30 × 10 ^−3^)	0.4	96.7	29
	3	0.6 (1.52 × 10 ^−3^)	1	92.9	13
5b	1	2.8 (8.09 × 10 ^−3^)	0.2	99.1	111
	2	1.5 (4.33 × 10 ^−3^)	0.4	98.4	63
	3	0.6 (1.73 × 10 ^−3^)	1	93.3	14
5c	1	2.9 (8.03 × 10 ^−3^)	0.2	97.1	34
	2	1.5 (4.15 × 10 ^−3^)	0.4	96.5	28
	3	0.6 (1.66 × 10 ^−3^)	1	83.5	5

* All experiments were conducted two times, and the data presented are an average of the obtained values.

Figure S3 Effect of KCI concentration on the removal efficiencies.

Figure S4 Molecular absorption spectra of (1) MO and (2) 4b: MO ion pairs in dichloromethane.

Figure S5 (A) Molecular absorption spectra of (1) 4b: MO ion pairs (2) 4a: MO ion pairs (3) 4c: MO ion pairs in dichloromethane. (B) Molecular absorption spectra of (1) 5b: MO ion pairs (2) 5a: MO ion pairs (3) 5c: MO ion pairs in dichloromethane.

Figure S6 Job’s method of continuous variation plot for the reaction of 4b salt with MO, [4b] = [MO] = 0.27 mmol/L.
