# Supplementary figures and images for: Tunable aryl alkyl pyrazolium tetrafluoroborate ionic liquids/salts: synthesis, characterization, and applications for removal of methyl orange from aqueous solution
Source: Turk J Chem. 2021 Sep 27;45(6):1988–96. doi: 10.3906/kim-2106-67 (PMC10734728; doi:10.3906/kim-2106-67)

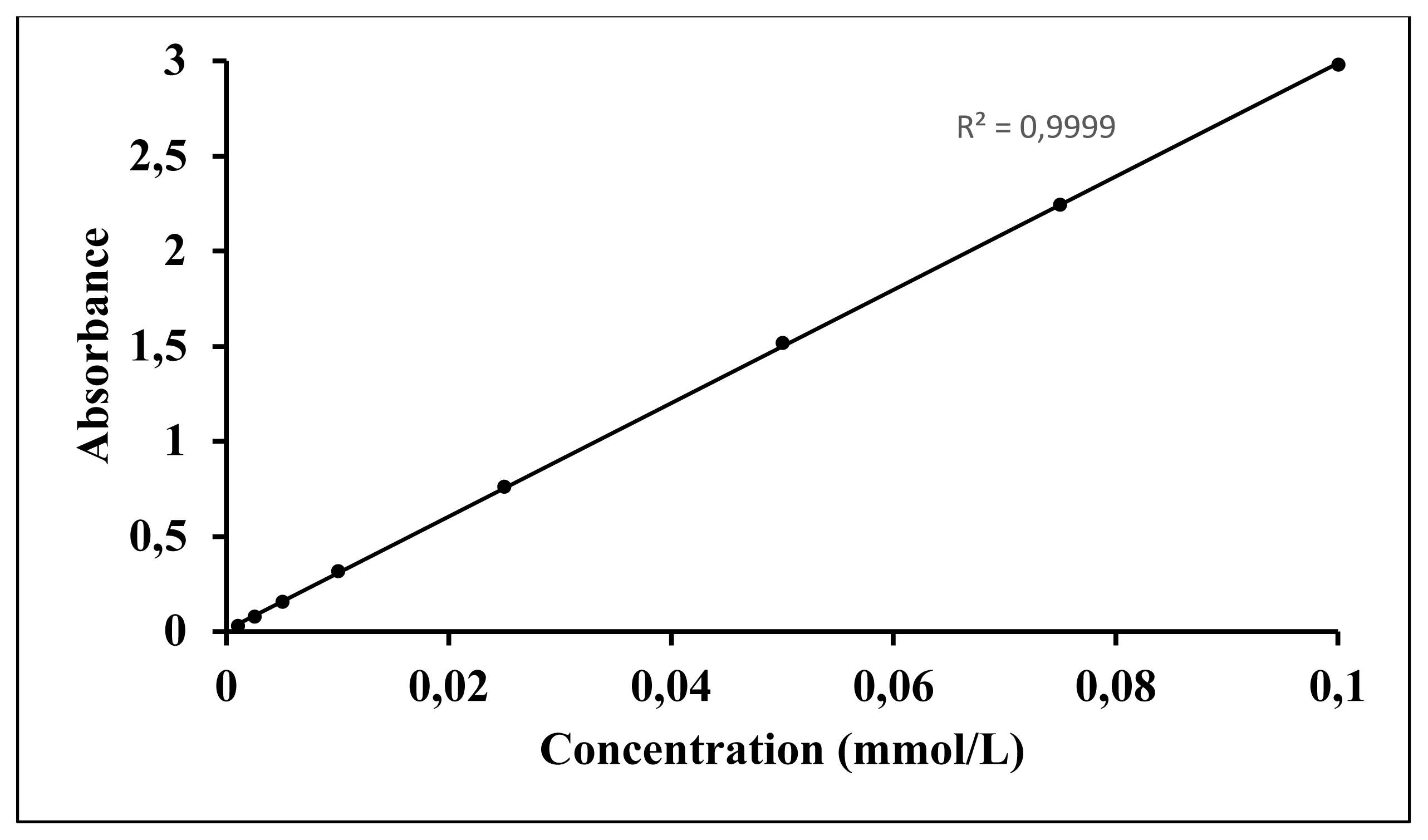

Supplement: Figure S1 — Calibration curve of methyl orange (0.1–0.001 mmol/L). [file turkjchem-45-6-1988s1.tif]

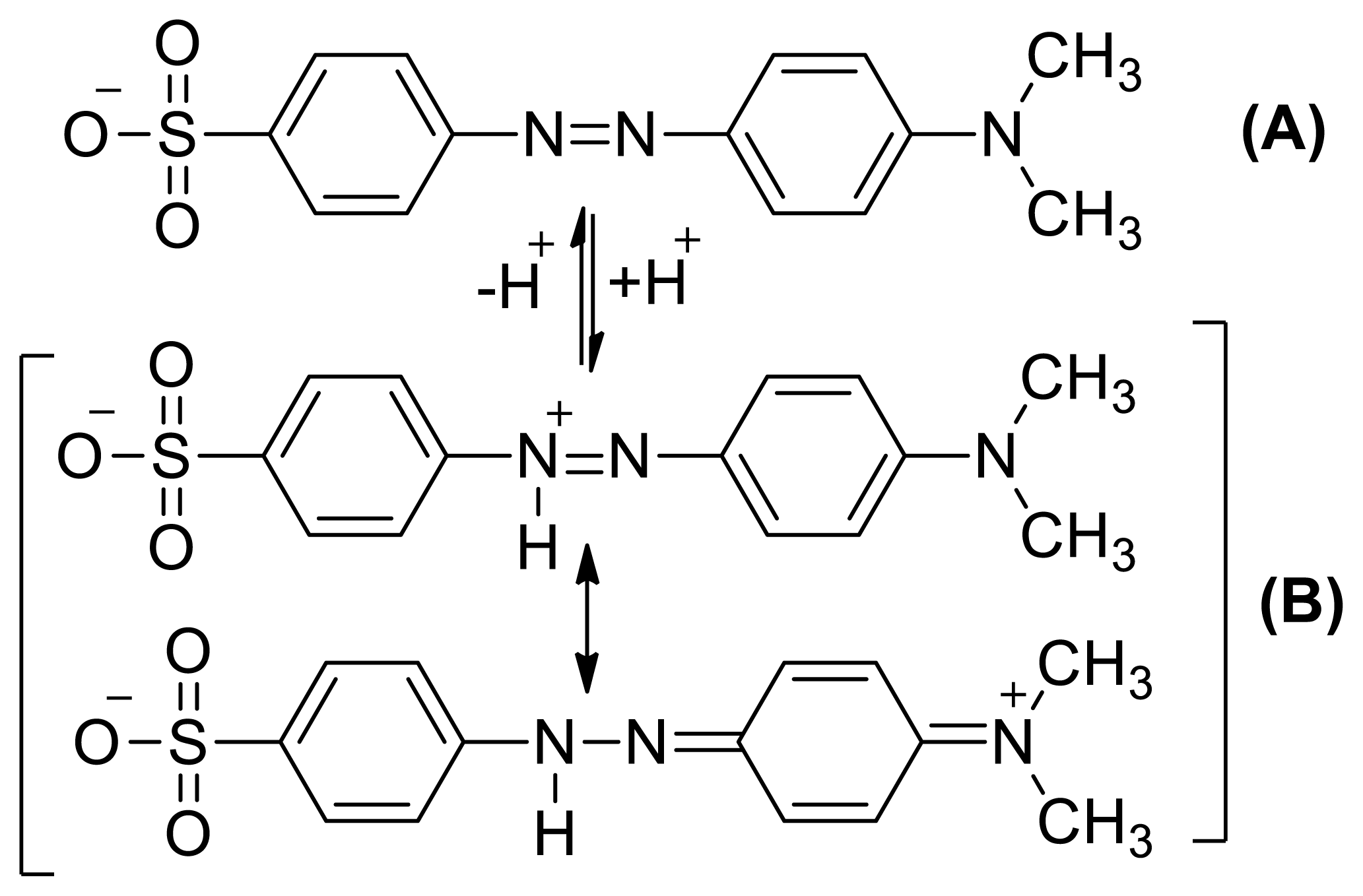

Supplement: Figure S2 — The main structures of MO in solutions (A) anionic form with pH above 3.8 (yellow) (B) zwitterionic form with pH below 3.8 (red). [file turkjchem-45-6-1988s2.tif]

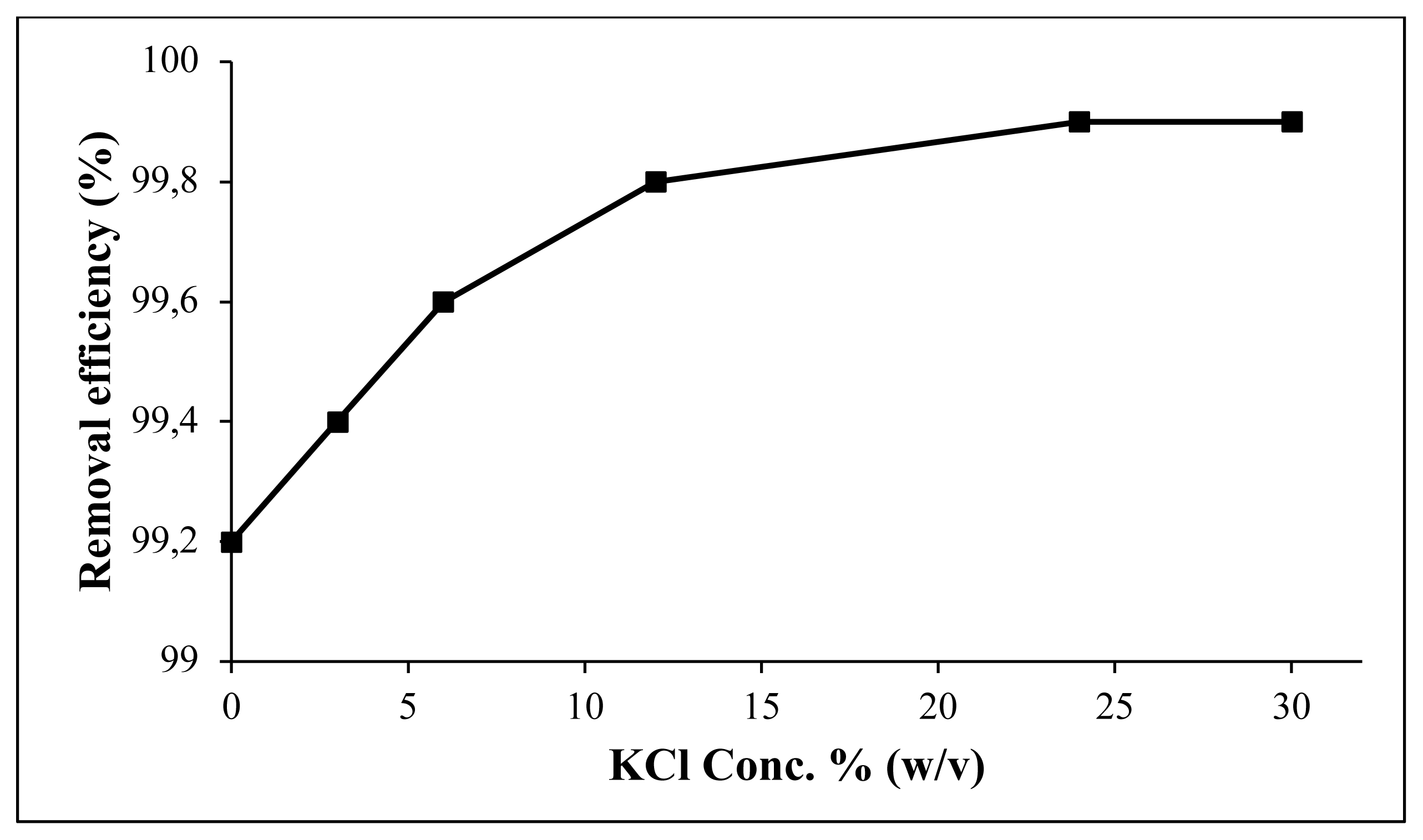

Supplement: Figure S3 — Effect of KCI concentration on the removal efficiencies. [file turkjchem-45-6-1988s3.tif]

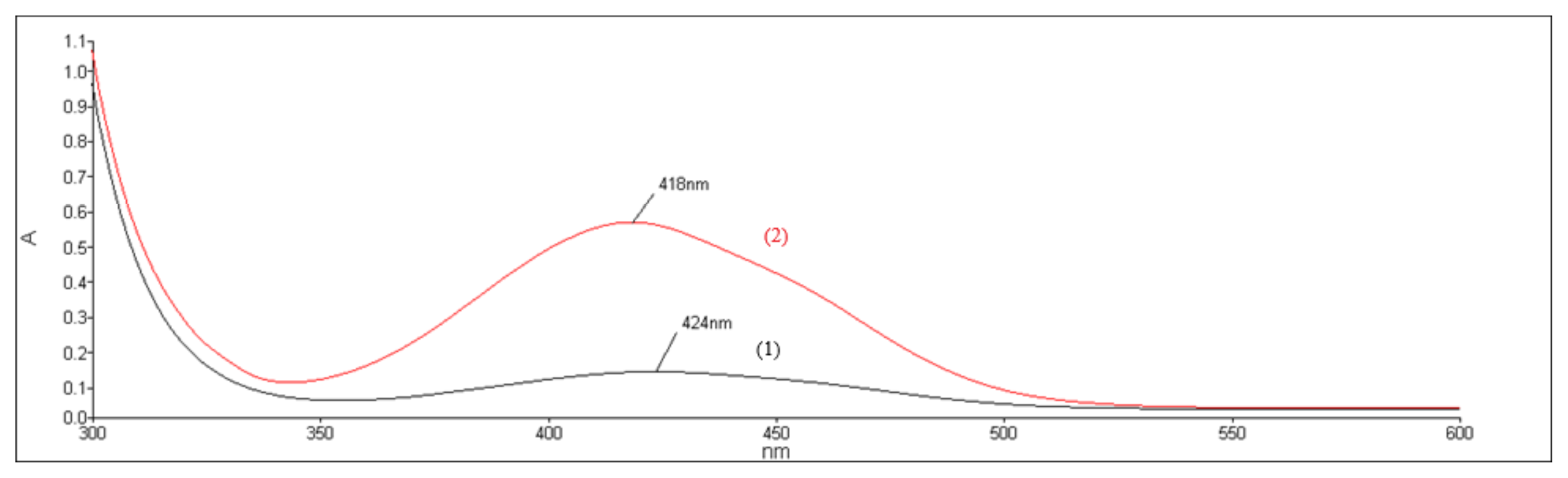

Supplement: Figure S4 — Molecular absorption spectra of (1) MO and (2) 4b: MO ion pairs in dichloromethane. [file turkjchem-45-6-1988s4.tif]

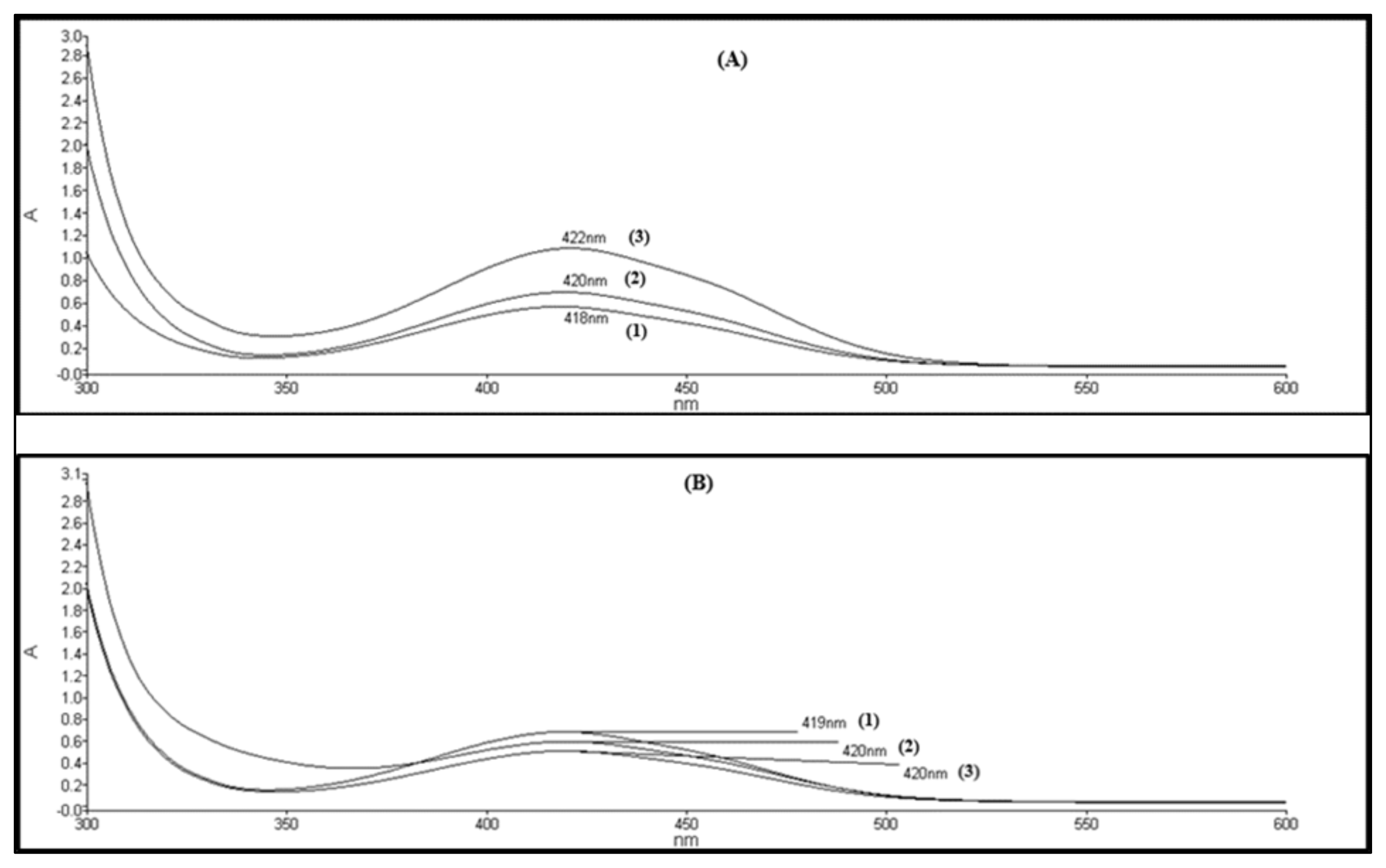

Supplement: Figure S5 — (A) Molecular absorption spectra of (1) 4b: MO ion pairs (2) 4a: MO ion pairs (3) 4c: MO ion pairs in dichloromethane. (B) Molecular absorption spectra of (1) 5b: MO ion pairs (2) 5a: MO ion pairs (3) 5c: MO ion pairs in dichloromethane. [file turkjchem-45-6-1988s5.tif]

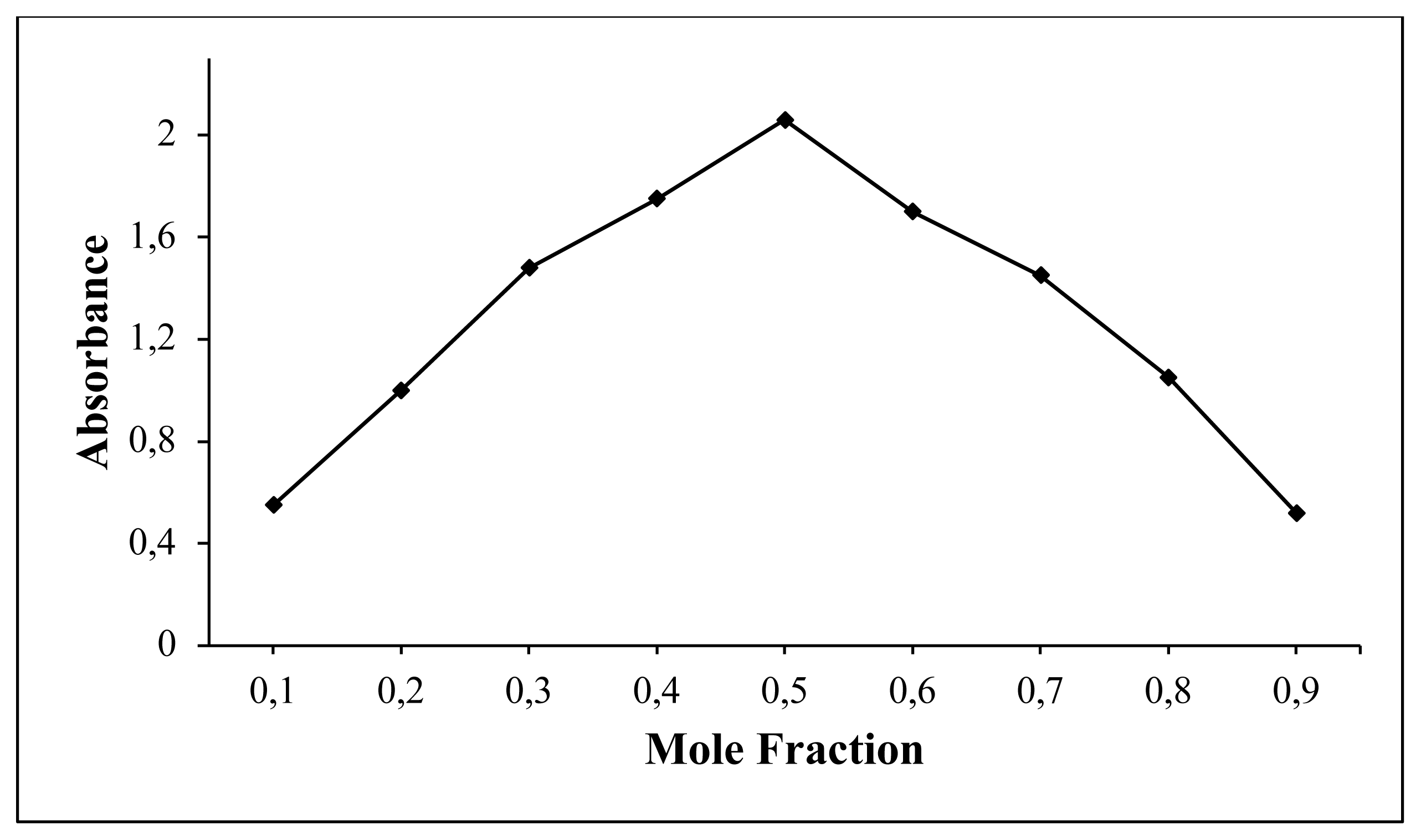

Supplement: Figure S6 — Job’s method of continuous variation plot for the reaction of 4b salt with MO, [4b] = [MO] = 0.27 mmol/L. [file turkjchem-45-6-1988s6.tif]
